# The Hippocampus and Neocortical Inhibitory Engrams Protect against Memory Interference

**DOI:** 10.1016/j.neuron.2018.11.042

**Published:** 2019-02-06

**Authors:** Renée S. Koolschijn, Uzay E. Emir, Alexandros C. Pantelides, Hamed Nili, Timothy E.J. Behrens, Helen C. Barron

**Affiliations:** 1 Wellcome Centre for Integrative Neuroimaging, University of Oxford, FMRIB, John Radcliffe Hospital, Oxford, OX3 9DU, UK; 2 School of Health Sciences, Purdue University, IN 47907, USA; 3 Department of Experimental Psychology, University of Oxford, 15 Parks Rd., Oxford OX1 3AQ, UK; 4 The Wellcome Centre for Neuroimaging, Institute of Neurology, University College London, London, WC1N 3BG, UK; 5 MRC Brain Network Dynamics Unit, University of Oxford, Mansfield Rd., Oxford OX1 3TH, UK

**Keywords:** memory, interference, associative learning, EI balance, inhibition, tDCS, GABA, hippocampus

## Abstract

Our experiences often overlap with each other, yet we are able to selectively recall individual memories to guide decisions and future actions. The neural mechanisms that support such precise memory recall remain unclear. Here, using ultra-high field 7T MRI we reveal two distinct mechanisms that protect memories from interference. The first mechanism involves the hippocampus, where the blood-oxygen-level-dependent (BOLD) signal predicts behavioral measures of memory interference, and representations of context-dependent memories are pattern separated according to their relational overlap. The second mechanism involves neocortical inhibition. When we reduce the concentration of neocortical GABA using trans-cranial direct current stimulation (tDCS), neocortical memory interference increases in proportion to the reduction in GABA, which in turn predicts behavioral performance. These findings suggest that memory interference is mediated by both the hippocampus and neocortex, where the hippocampus separates overlapping but context-dependent memories using relational information, and neocortical inhibition prevents unwanted co-activation between overlapping memories.

## Highlights

• Memory interference is mediated by both the hippocampus and neocortical inhibition
• In the hippocampus, overlapping memories are separated using relational information
• In neocortex, inhibition protects overlapping memories from interference
• By reducing neocortical GABA with brain stimulation, memory interference increases

## Teaser

Koolschijn et al. reveal two distinct mechanisms that protect overlapping memories from interference in the human brain. One involves separating memories in the hippocampus. The other involves neocortical inhibition, which protects against memory interference unless GABA is reduced using brain stimulation.

## Introduction

Our decisions and actions are often guided by past experiences that overlap with each other in content or sensory information. To ensure that interference between related or overlapping experiences is minimized, a stable memory storage system is critical. However, the precise physiological mechanism that supports stable memory storage in the absence of memory interference remains unclear.

One way to minimize memory interference is to separate stored information using contextual representations (McClelland et al., 1995, Norman and O’Reilly, 2003, Shapiro and Olton, 1994). Behavioral data in humans provides supporting evidence for this mechanism, as contextual cues help mitigate memory interference between two lists of paired associates (Bilodeau and Schlosberg, 1951). At the neural level, anticorrelated firing patterns for opposing contexts can be observed in the hippocampal output regions (Butterly et al., 2012, McKenzie et al., 2014). These contextual representations likely reflect the natural consequence of pattern separation, a competitive mechanism supported by the architecture of the hippocampus that orthogonalizes representations of overlapping memories (Yassa and Stark, 2011). However, it remains unclear how information within contextual representations is organized. One possibility is that contextual information is organized in a manner that reflects the relational or configural structure of memory elements (Cohen and Eichenbaum, 1993, Sutherland and Rudy, 1989). Consistent with this suggestion, here we hypothesize that the hippocampus helps protect against memory interference by separating elements of a partially overlapping memory according to their relational similarity. Thus, elements that have different relational positions across two overlapping but context-dependent memories are maximally separated.

In addition, an alternative way to protect stored memories from interference involves using inhibition. Following learning, new information is thought to be stored in the brain via modification in the strength of excitatory connections (Hebb, 1949, Nabavi et al., 2014, Song and Abbott, 2001). In turn, these newly modified excitatory connections are opposed by equivalent changes in the strength of inhibitory connections (Barron et al., 2016a, Froemke et al., 2007, Vallentin et al., 2016, Vogels et al., 2011). This allows excitatory-inhibitory (EI) balance to be maintained despite new learning (Froemke et al., 2007, Haider et al., 2006, Okun and Lampl, 2008, Wehr and Zador, 2003), and ensures that memories lie dormant unless EI balance is disturbed (Barron et al., 2016a, Jacobs and Donoghue, 1991, Vallentin et al., 2016). Here, we hypothesize that the inhibitory component of a memory, otherwise termed the inhibitory engram (Barron et al., 2017), protects memories from interference by preventing runaway excitation.

Consistent with this hypothesis, context-dependent behavior in rodents is accompanied by modulation of neocortical interneurons (Kuchibhotla et al., 2017), while, in humans, an increase in neocortical GABA relative to glutamate accompanies overlearning, a process known to protect memories from interference (Shibata et al., 2017). Clinical investigations also support a key role for inhibitory regulation of memory expression, as impaired GABAergic regulation can readily account for delusions and hallucinations reported in schizophrenia (Vogels and Abbott, 2007, Yizhar et al., 2011). Thus, by gating memory expression (Barron et al., 2016a, Barron et al., 2017, Vogels and Abbott, 2009), inhibitory engrams may play a critical role in preventing unwanted interference between overlapping memories.

Here, we investigate the role of both the hippocampus and neocortical inhibition in protecting against memory interference. First, we test the hypothesis that contextual representations in the hippocampus are organized using a relational code, thus separating competing memories according to behaviorally relevant information. Second, we test the hypothesis that neocortical inhibition protects overlapping memories from interference.

To this end, we designed a task that required participants to encode two overlapping but context-dependent memories across two consecutive days. On the third day, interference between the two memories was measured using ultra-high field 7T MRI. In the hippocampus, we observed an increase in blood-oxygen-level-dependent (BOLD) signal during opportunities for memory interference, which predicted subsequent behavioral performance. In addition, representations of stimuli that had different relational positions across the two overlapping but context-dependent memories were maximally separated. Then, to investigate the role of neocortical inhibition in protecting memories from interference, halfway through the scan, we manipulated the concentration of neocortical GABA using brain stimulation and re-assessed evidence for memory interference. The drop in neocortical GABA induced by brain stimulation predicted an increase in neocortical memory interference, which in turn predicted deficits in behavioral performance. Together these results suggest that memory interference is mediated by two distinct mechanisms: a hippocampal mechanism in which contextual representations are organized according to behaviorally relevant relationships, and a neocortical mechanism in which inhibition protects overlapping memories from unwanted co-activation.

## Results

### Associative Learning and Experimental Design

**Figure 1:**
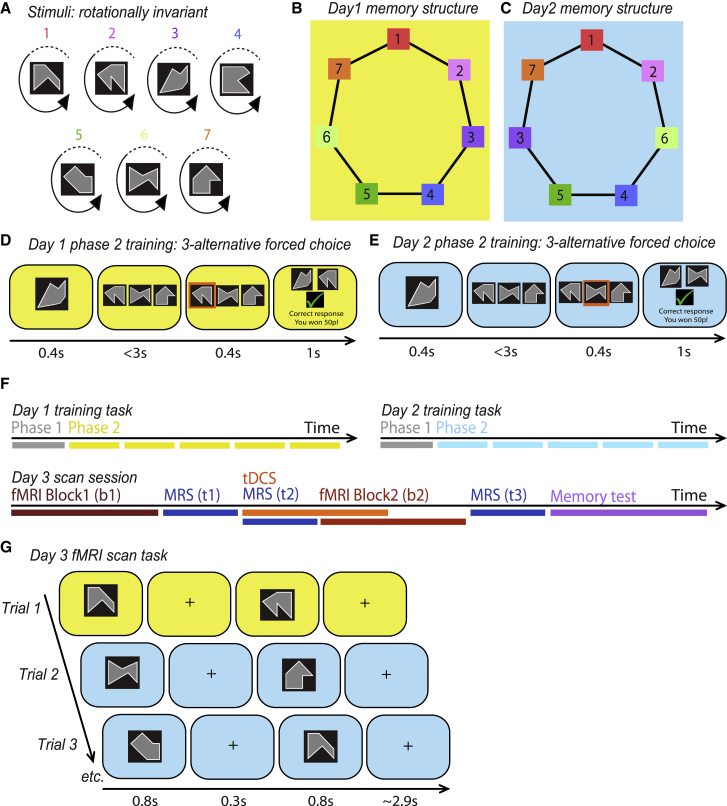
Experimental Design (A) Seven stimuli were used for the experiment, where each stimulus was an abstract shape which could appear in four possible rotations. (B–E) Example experimental protocol: (B) on day 1, participants learned seven associations between pairs of the seven stimuli shown in (A), and a contextual cue was provided using a yellow background. The associations could be arranged in a ring structure, although this was not explicitly shown to participants. (C) On day 2, participants learned seven associations between pairs of the seven stimuli. Four of the associations were different from those learned on day 1 as the position of stimuli 3 and 6 were swapped. A contextual cue was provided using a blue background, a different color from that used on day 1. To learn the associations on day 1 (D) and day 2 (E), participants performed a 3-alternative forced choice task where the appropriate background color was used to provide a contextual cue. (F) Schematic showing protocol used across all 3 days of the experiment. On day 1 and day 2, participants completed phase 1 (Figures S1A and S1B) and at least 5 blocks of phase 2 (Figures 1D and 1E) of the training task. On day 3 of the experiment, participants started with block 1 (“b1”) of the scan task, as shown in Figure 1G, before Magnetic Resonance Spectroscopy (MRS) measurements (“t1”) were taken to estimate baseline measures of 19 different metabolites. Anodal tDCS was then applied for a total of 20 min to induce EI imbalance, with MRS measurements (“t2”) taken during the first 10 min, before block 2 (“b2”) of the scan task commenced. After block 2 of the scan task, a third set of MRS measurements (“t3”) were obtained before participants exited the scanner to perform a surprise memory test. “b” indicates block of fMRI acquisition. “t” indicates time point of MRS acquisition. (G) During the scan task, participants observed pairs of stimuli presented consecutively against either a yellow or blue background. All possible pairs of stimuli were presented in a random order.

On day 1 of the experiment, participants learned a set of associations between seven rotationally invariant abstract stimuli (Figure 1A), which together formed “memory 1”. Within memory 1, each stimulus was associated with two other stimuli, giving seven bidirectional associations in total. The set of associations could be arranged into a ring structure (Figure 1B), although this was never made explicit to the participants. Instead, participants were instructed to learn the associations using a three-alternative forced choice task (Figures 1D, S1A, and S1C; see STAR Methods). Rotationally invariant abstract stimuli (Figure 1A) were used so that we could later make precise predictions about the brain region sensitive to the learned associations (Barron et al., 2016a).

On day 2 of the experiment, participants learned a second set of bidirectional associations between the same seven abstract stimuli (Figure 1A), which together formed “memory 2”. As in memory 1, each stimulus in memory 2 was associated with two other stimuli (Figure 1C). Participants again learned these associations using the three-alternative forced choice task (Figures 1E, S1B, and S1D). Critically, the relational position of the seven stimuli within the ring differed between memory 1 and memory 2, as the positions of stimuli 3 and 6 were switched (Figures 1B and 1C). Consequently, four of the seven associations in memory 2 were different from those in memory 1, while three associations remained the same. To help participants distinguish between memory 1 and memory 2, contextual cues were used, consisting of a unique background color (yellow or blue, randomized across participants) (Figures 1D and 1E) and a time interval of approximately 24 hr between learning sessions (Figure 1F).

Thus, memory 1 and memory 2 included the same stimuli but had different relational structures. The difference in relational structure was designed to ensure that a subset of associations across memory 1 and 2, those containing stimuli 3 or 6, were different, while the remaining associations were matched. We predicted that associations containing elements 3 or 6 were susceptible to memory interference, where memory interference manifests as recall of a relational neighbor from the alternative, inappropriate memory. Meanwhile, the matched portion of the two memories provided the necessary control. The experimental design, therefore, included precise and controlled markers of memory interference that could be assessed at both a behavioral and neural level.

### Hippocampus Mediates Memory Interference Using Context-Dependent Relational Codes

To identify the physiological mechanisms that protect memories from interference, we first considered the contribution made by the hippocampus. First, we sought to show evidence for pattern separation between memory 1 and 2 in the hippocampus, thus building on prior evidence (Bonnici et al., 2012, Huffman and Stark, 2014, Yassa and Stark, 2011). Second, we assessed the organization of contextual representations that reflect the output of pattern separation. In accordance with the idea that the hippocampus organizes representations according to a relational code (Eichenbaum, 2004, Nadel, 2008), we hypothesized that elements that have different relational positions across two overlapping but context-dependent memories are maximally separated. For the paradigm implemented here, we predicted that representations of stimuli 3 and 6 would show maximum pattern separation across memory 1 and memory 2.

To test these predictions, on day 3 of the experiment we used fMRI to measure the BOLD response to the associative memories learned in memory 1 and memory 2 (Figure 1F). On each trial of the scan task, a pair of stimuli was presented on either a yellow or a blue background to provide a contextual cue for memory 1 or memory 2 (Figure 1G; see STAR Methods). We controlled for potential confounds introduced by expectation suppression (Summerfield et al., 2008) by ensuring that each possible pair of stimuli was presented equally often in a fully randomized order. To ensure participants paid close attention to the stimuli presented during the scan, participants were instructed to detect “odd-ball” stimuli, which were not part of the seven experienced during training. To improve the signal to noise ratio (SNR) of the fMRI data in brain regions for which we had strong prior hypotheses, we restricted the fMRI sequence to a partial volume, thus increasing the number of measurements acquired (for example partial volume see Figures S2A–S2C).

**Figure 2:**
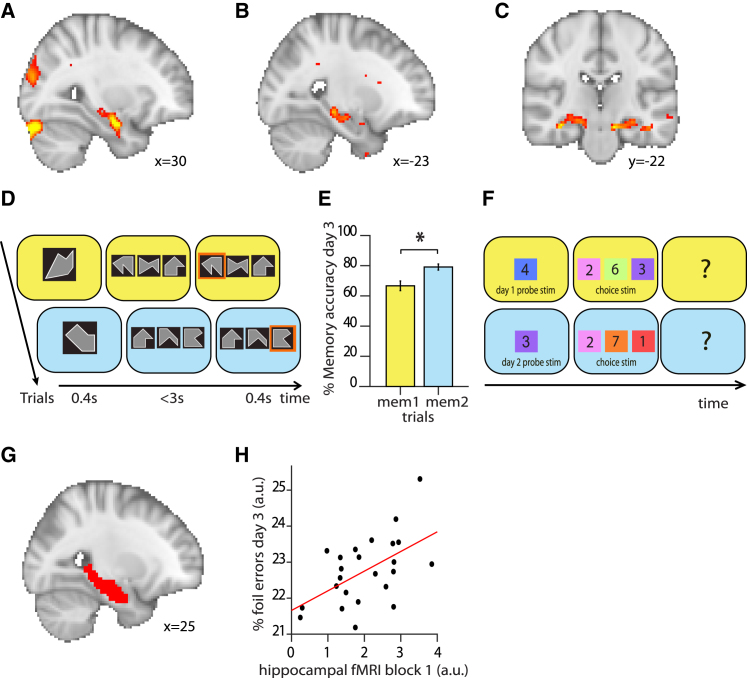
The Hippocampus Mediates Memory Interference (A–C) Hippocampal BOLD signal was higher on trials where there was opportunity for memory interference (i.e., when trials include stimuli 3 or 6 that have a different relational position between memory 1 and memory 2; contrast estimated across block 1 and block 2 of the fMRI task, thresholded at p < 0.01 for visualization). Hippocampal BOLD signal was significantly higher in right hippocampus (t_23_ = 4.34, p = 0.015, FWE peak-corrected using a small-volume correction (SVC) method, (A), while a similar trend was observed in the left hippocampus (t_23_ = 3.66, p = 0.056, FWE peak-corrected using a SVC method, (B). (C) Hippocampal BOLD signal across both hippocampi, for visualization. (D) After exiting the scanner on day 3, participants performed a surprise memory test. On each trial, participants were presented with a probe stimulus with the background color providing the contextual cue. They were then presented with three option stimuli and were required to choose the stimulus correctly paired with the probe stimulus in the absence of feedback. (E) Memory accuracy on the surprise memory test (mean, ± SEM), for memory 1 (“mem1”) and memory 2 (“mem2”). The more recent associations in memory 2 were remembered more accurately than those in memory 1 (paired t test: t_25_ = 3.99, p < 0.001). (F) Foil trials on the surprise memory test shown in (D) were identified as those trials where one of the three stimuli was incorrect given the current context but correct in the alternative context. (G) Hippocampal ROI across both right and left hippocampi, used to perform SVC method in (A) and (B), to extract parameter estimates in (H), and to extract activity patterns for Representational Similarity Analysis (RSA) in Figure 3. (H) The hippocampal BOLD response to trials where there was opportunity for memory interference in block 1 (Figure 1F) predicted the number of foil errors, a behavioral index for memory interference, on the post-scan surprise memory test (Pearson’s correlation: r_23_ = 0.54, p = 0.006, after accounting for differences in learning, see STAR Methods). “a.u.” refers to “arbitrary units.”

Across the two fMRI task blocks (“block 1” and “block 2”), we observed an increase in the hippocampal BOLD signal to pairs of stimuli that had a different relational position across memory 1 and memory 2 (i.e., pairs of stimuli that included stimuli 3 and 6), relative to pairs of stimuli that had the same relational position across both memories (Figures 2A–2C: contrast of interest; right hippocampus: t_23_ = 4.34, p = 0.015, peak-level Family-Wise Error (FWE) corrected using small volume correction (SVC), Figure 2A; left hippocampus: t_23_ = 3.66, p = 0.056, peak-level FWE corrected using SVC, Figure 2B). Therefore, the hippocampal BOLD signal increased on trials where there was opportunity for interference between the two memories.

Next, we asked whether this hippocampal BOLD signal could predict behavioral measures of memory interference. After the scan session, participants performed a surprise memory test and we assessed recall accuracy for all seven associations within memory 1 and memory 2. The memory test involved the three-alternative force choice task used during training, but now in the absence of feedback (Figure 2D). On average, participants correctly recalled the appropriate association on 72.9% of trials, showing higher accuracy for more recent memories (paired-sample t test, t_25_ = 3.99, p < 0.001, Figure 2E). In addition to participants’ overall memory accuracy, behavioral memory interference was quantified using participants’ performance on “foil trials,” namely those trials where the choice stimuli included the stimulus that was correct given the contextual background cue, but also a “foil” stimulus that would be correct in the alternative memory (Figure 2F). The percentage of foil errors made by a participant corresponded to the percentage of foil trials where the foil stimulus was chosen rather than the correct stimulus. Thus, the percentage of foil errors characterized the extent to which participants recalled associative memories learned in context 2 when in context 1 (or vice versa), providing a behavioral measure of memory interference that reflects inappropriate overgeneralization across contexts. Across participants, we observed a positive relationship between the hippocampal BOLD signal and the percentage of foil errors (hippocampal BOLD signal to pairs of stimuli that included stimuli 3 or 6 minus all other pairs in block 1, from ROI shown in Figure 2G, versus percentage of foil errors: r_23_ = 0.54, p = 0.006, Figure 2H, after accounting for differences in learning; see STAR Methods).

**Figure 3:**
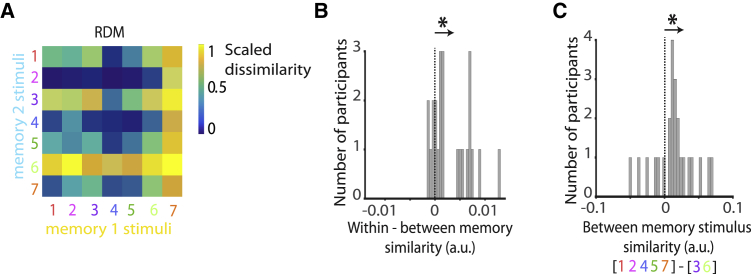
Hippocampal Representations Show Evidence for Contextual Pattern Separation, Organized by Relational Overlap For each trial in the scan task, the pattern of BOLD activity across voxels was extracted from the hippocampus (Figure 2G), and representational dissimilarity between all trials containing each of the 7 stimuli was assessed using representational similarity analysis (RSA). For example, representations of stimulus 1 in memory 1 included all pairs of stimuli shown on a yellow background that included stimulus 1 (i.e., 1–1, 1–2, … 1–7). (A) Representational dissimilarity matrix (RDM) showing the average representational dissimilarity between stimuli in memory 1 and memory 2, averaged across all participants, rank transformed and scaled into [0–1] for visualization. (B) The RDM for each participant was correlated with a model RDM shown in Figure S2D to test evidence for pattern separation of hippocampal representations by memory. Across participants, significant representational similarity within memory 1/2 versus between memory 1 and memory 2 was observed (Wilcoxon sign rank test: Z_23_ = 3.34, p = < 0.001), even if stimuli 3 and 6 were excluded (see Figure S2G). Note: the dissimilarity of a trial to itself was excluded from the analysis. “a.u.” refers to “arbitrary units.” (C) The RDM for each participant was correlated with a model RDM shown in Figure S2E to test evidence for increased representational dissimilarity of stimuli that had different relative position across memory 1 and memory 2 (stimuli 3 and 6). Across participants, significantly greater representational dissimilarity between memory 1 and memory 2 was observed for stimuli 3 and 6 compared to all other stimuli (Wilcoxon sign rank test: Z_23_ = 2.26, p = 0.024). “a.u.” refers to “arbitrary units.”

While these results suggest that the hippocampus may play a key role in mediating memory interference, they leave open the nature of the hippocampal code. If the hippocampus uses contextual representations to pattern separate overlapping memories, then representations of stimuli within memory 1 should be more similar to representations of other stimuli within memory 1 compared to representations of stimuli in memory 2. To test this hypothesis, we extracted the pattern of activity across voxels in a hippocampal ROI (Figure 2G) for each trial in both block 1 and block 2. Then, we used representational similarity analysis (RSA) to quantify the representational similarity between memory 1 and memory 2 for each of the seven stimuli using the Mahalanobis distance (Figure 3A; see STAR Methods). We observed higher representational similarity within versus between memory (Figure 3B; Wilcoxon sign rank test across the group: Z_23_ = 3.34, p < 0.001; Model RDM: Figure S2D). Consistent with previous literature (Bonnici et al., 2012, Huffman and Stark, 2014, Yassa and Stark, 2011), this suggests that stimulus representations in the hippocampus were pattern separated according to contextual information.

Having shown evidence for pattern separation between memory 1 and memory 2, we next asked how contextual representations for memory 1 and memory 2 are organized. We predicted that representations of stimuli within memory 1 and memory 2 are pattern separated in a manner that reflects the overlap in their relational positions. Given the structure of the learned information, the relational position of each stimulus was defined by its neighbors within the ring structures (Figures 1B and 1C). Therefore, only stimuli 3 and 6 had different relational positions across memory 1 and memory 2. Across memory 1 and memory 2, representational dissimilarity was higher for stimuli 3 and 6 relative to all other stimuli (stimuli 1, 2, 4, 5, and 7) (Figure 3C; Wilcoxon sign rank test across the group: Z_23_ = 2.26, p = 0.024; Model RDM: Figure S2E). Notably, for this cross-memory comparison, only the background color of the stimuli changed. This result suggests that contextual representations are organized according to the relational overlap of competing memories, where memory elements that have a different relational position across two memories are represented using more distinct neural codes.

### Manipulating Neocortical EI Balance to Measure the Effect of Inhibition on Memory

**Figure 4:**
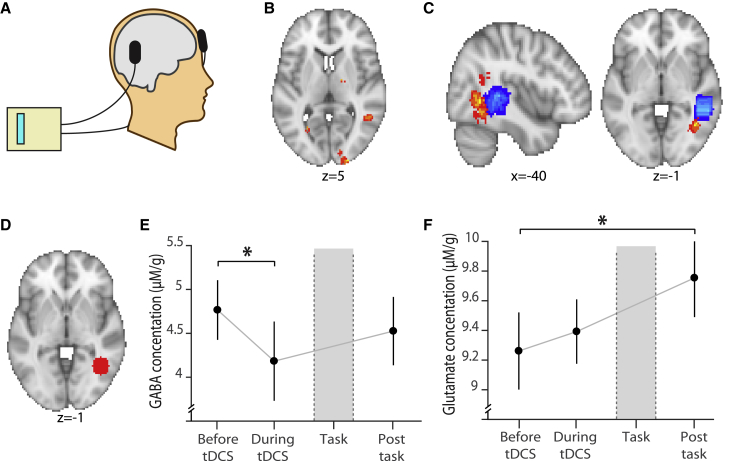
Manipulating Neocortical EI Balance using Brain Stimulation A) After the first scan task (block 1) and while participants lay in the scanner, anodal tDCS was applied to the aLOC, with the cathodal electrode placed over the contralateral supraorbital ridge. (B) Previously published data (Barron et al., 2016a) shows re-expression of associative memories during application of anodal tDCS. This aLOC region was the target location for the anodal tDCS electrode in the current experimental protocol. Orientation: neurological. (C) Blue: average location of the MRS voxel across participants. Red: average location of the anodal tDCS electrode across participants, projected into the brain (see STAR Methods). Orientation: neurological. (D) 10mm radius sphere defined around the peak tDCS electrode location for all participants (see STAR Methods), used as a region of interest and for small volume correction. Orientation: neurological. (E) MRS was used to quantify the concentration of GABA relative to total Creatine at three time points indicated in Figure 1F (shown: mean ± SEM). A significant reduction in relative GABA was observed during tDCS (“Before tDCS” – “During tDCS,” t_19_ = 2.32, p = 0.016). (F) MRS was used to quantify the concentration of glutamate relative to total creatine at three time points indicated in Figure 1F (shown: mean ± SEM). A significant increase in relative glutamate was observed after the second scan-task (“Post-task” – “Before tDCS,” t_19_ = 2.60, p = 0.018).

Having characterized a role for the hippocampus in mediating memory interference, we next asked whether inhibition in the neocortex also plays a key role. In neocortex, associative memories appear to be stored by excitatory connections that are later balanced by matched inhibition (Barron et al., 2016a, Froemke et al., 2007, Vallentin et al., 2016, Vogels and Abbott, 2009). Therefore, by day 3 of the experiment, we expected neocortical representations of memory 1 and memory 2 to be stored in balanced EI ensembles. However, if neocortical inhibition plays a critical role in protecting overlapping memories from unwanted interference then it should be possible to induce interference by reducing inhibitory tone. To test this prediction, in the second half of the day 3 scan session we applied non-invasive anodal transcranial direct current stimulation (tDCS) (Figure 4A), a tool previously used to induce a transient reduction in the concentration of neocortical GABA (Barron et al., 2016a, Kim et al., 2014, Stagg et al., 2009) and to unmask otherwise silent neocortical associative memories (Figure 4B) (Barron et al., 2016a).

Direct current stimulation increases cortical excitability, such that neuronal firing rates increase (Bindman et al., 1962) along with remote motor evoked potentials measured using transcranial magnetic stimulation (TMS) (Nitsche et al., 2005). After stimulation, the increase in cortical excitability is sustained for minutes to hours (Bindman et al., 1962) via a protein synthesis dependent process (Nitsche and Paulus, 2000), which can be used to enhance learning (Jacobson et al., 2012) and recovery from stroke (Hummel and Cohen, 2006). Critically, the mechanism responsible for this increase in cortical excitability appears to involve a reduction in the concentration of available GABA, as evidenced by *in vivo* spectroscopic measurements (Barron et al., 2016a, Kim et al., 2014, Stagg and Nitsche, 2011, Stagg et al., 2009).

Taking advantage of this non-invasive tool, we placed the anodal tDCS electrode over anterior Lateral Occipital Complex (aLOC) to induce variance in EI balance, in the target brain region known to encode learned associations for rotationally invariant shapes (Barron et al., 2016a) (Figure 4B). The cathodal electrode was placed over the contralateral supraorbital ridge (Figures 4A and S5A–S5D). Brain stimulation was applied immediately before participants performed a second run of the scan task (block 2, Figure 1F). Before, during, and after brain stimulation, we used Magnetic Resonance Spectroscopy (MRS) to rapidly measure the concentration of 19 different neural metabolites (Figure 1F), including GABA and glutamate, from a 2×2×2cm^3^ voxel placed just anterior of the anodal electrode (Figure 4C). The concentration of each neural metabolite was assessed relative to the concentration of total Creatine (Cr), a suitable reference metabolite. Consistent with previous literature, application of anodal tDCS was accompanied by a significant reduction in the concentration of GABA relative to baseline (GABA:Cr for “baseline” > “tDCS,” t_19_ = 2.32, p = 0.016, Figures 4E and S3E). As a consequence, block 2 of the scan task was performed in a state of EI imbalance, where excitation outweighed inhibition. The reduction in GABA was not sustained to the period after the task (GABA:Cr for “baseline” > “post-task,” t_19_ = 0.83, p = 0.414, Figure 4E). In addition to this change in GABA, we also observed a significant increase in the concentration of glutamate but only after the second task session (glutamate:Cr for “baseline” < “post-tDCS,” t_19_ = 2.60, p = 0.018; Figures 4F and S3F). This change in glutamate may be attributed to participants performing block 2 of the scan task and doing so in a state of EI imbalance. See Table S1 for list of all measured metabolites.

### Measuring Associative Memories Using Cross-Stimulus Suppression

**Figure 5:**
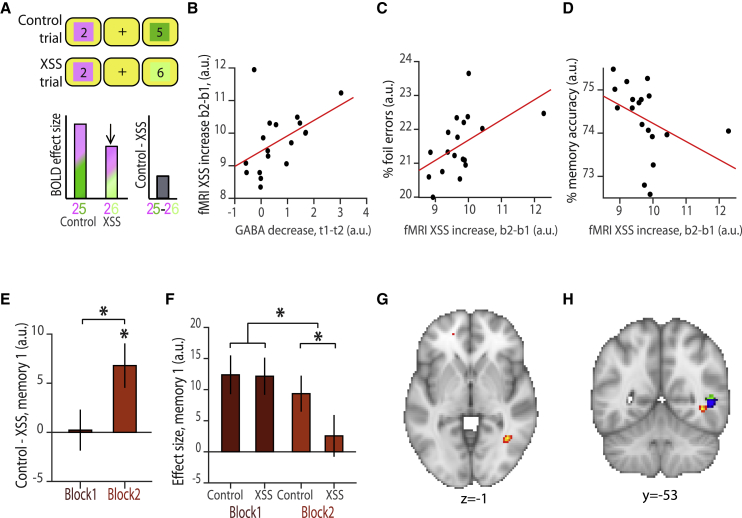
Memory Interference for More Remote Memories Increases with Brain Stimulation In all panels, “a.u.” refers to “arbitrary units”; XSS indicates cross-stimulus suppression; “b” indicates block of fMRI acquisition, as shown in Figure 1F; “t” indicates “time point” of MRS measurement acquisition, as shown in Figure 1F. (A) When participants performed the scan task in EI imbalance, we predicted an increase in XSS on trials where participants observed pairs of stimuli that were unassociated in the current context but directly associated in the alternative context, relative to control trials where participants observed pairs of stimuli that were unassociated in both contexts. This difference between control and XSS trials could be measured using the BOLD signal and was used as an index for neural memory interference. (B) Across participants, the decrease in relative GABA concentration observed during tDCS (“Before tDCS” – “During tDCS,” Figure 4E) positively predicted the increase in neural memory interference measured using fMRI cross-stimulus suppression (control – XSS for block 2 – block 1, memory 1 and memory 2) (Spearman correlation: r_17_ = 0.55, p = 0.021, after accounting for changes in glutamate; see STAR Methods). (C) Across participants, the increase in cross-stimulus suppression used to measure memory interference (control – XSS for block 2 – block 1) positively predicted the percentage of foil errors participants made on the surprise memory test on day 3 (Spearman correlation: r_17_ = 0.58, p = 0.013, after accounting for differences in learning and changes in relative GABA and glutamate). (D) Across participants, the increase in cross-stimulus suppression used to measure memory interference (control – XSS for block 2 – block 1, Figure 5A) negatively predicted average memory accuracy on the surprise memory test on day 3 (Spearman correlation: r_17_ = −0.67, p = 0.003, after accounting for differences in learning and changes in relative GABA and glutamate). (E) Within an ROI defined from the peak average tDCS electrode location shown in Figure 4C, extracted parameter estimates for memory 1 (shown: mean ± SEM) revealed a significant increase in the fMRI cross-stimulus suppression measure for memory interference (control – XSS, as shown in (A)) from block 1 to 2 and during block 2 alone (control – XSS for block 2 – block 1: t_23_ = 3.05, p = 0.006; control – XSS for block 2: t_23_ = 3.00, p = 0.006). (F) Extracted parameter estimates from (E) split into the control and XSS conditions, as described in (A) (shown: mean ± SEM). (G) T-statistic map for cross-stimulus suppression index for neural memory interference during block 2 between unassociated stimuli in memory 1 that are directly associated in memory 2 relative to pairs of stimuli that are unassociated in both memories (threshold at p < 0.01 uncorrected for visualization). Orientation: neurological. (H) Illustrating the anatomical proximity between the effects shown in Figures 5G (red) and S4G (blue) and previously acquired dataset shown in Figure 4B (green). Orientation: neurological.

To test whether aLOC inhibition protects memories from interference, we assessed evidence for neural memory interference during the transient period of induced EI imbalance. To measure memory interference in aLOC, we sought to index co-activation between representations for different memory elements. We took advantage of fMRI repetition suppression, which relies on the fact that neurons show a relative suppression in their activity in response to repeated presentation of a stimulus to which they are sensitive (Miller et al., 1991, Sawamura et al., 2005). While typically used to access sub-voxel representations for single stimuli (Grill-Spector et al., 2006), “cross-stimulus” suppression can be used to index the relative co-activation or overlap between representations coding for two different stimuli (Barron et al., 2016b). We contrasted the BOLD response for each pair of stimuli where suppression was expected against the BOLD response to a control pair where suppression was not expected, thus controlling for attentional effects (Figures 5A and S4A). The ring topology of memory 1 and memory 2 provided an efficient way to ensure that each stimulus contributed to both trials where suppression was expected (directly associated stimuli in one or both contexts) and control trials where suppression was not expected (stimuli separated by up to three associations in both contexts).

We first replicated our previous findings (Barron et al., 2016a), showing that cross-stimulus suppression increases during anodal transcranial direct current stimulation (tDCS) between directly associated stimuli that remain the same across memory 1 and 2 (Figure S4C). Furthermore, this increase in cross-stimulus suppression can be predicted by the relative decrease in GABA concentration (Figure S4H). This implies that inhibition in aLOC acts to quench memory expression of recently acquired associative memories, but during periods of EI balance these otherwise dormant memories are re-expressed. Interestingly, the extent to which associative memories were re-expressed during EI imbalance was significant for memory 2 but not memory 1 alone (Figures S4D–S4G). This difference between recent and more remote memories may in part be explained by a difference in the strength of associations in memory 1 compared to memory 2, which could be observed at a behavioral level (paired-sample t test, t_25_ = 3.99, p < 0.001, Figure 2E), even for associations that remained the same across memory 1 and memory 2 (paired-sample t test, t_25_ = 2.16, p = 0.040, Figure S4I).

### Memory Interference Increases during Periods of EI Imbalance

Having replicated our previous findings (Figure S4, Barron et al., 2016a), we went on to investigate whether neocortical inhibition plays a critical role in protecting against memory interference. Capitalizing on the inter-subject variability to the anodal tDCS manipulation (Figure 4E), we predicted a two-way relationship between the drop in relative GABA, our neural measures of memory interference and behavioral measures of memory interference: if neocortical inhibition protects memories from interference, the drop in relative GABA should predict neural measures of memory interference, which should in turn predict behavioral measures of memory interference.

As neural memory interference manifests as activation of a relational neighbor from the alternative, inappropriate memory, we sought to index this unwanted activation using cross-stimulus suppression. To this end, we identified trials during the scan task where participants were shown two stimuli that were unassociated given the memory indicated by the contextual cue, but directly associated in the alternative memory. During periods of EI imbalance, we predicted an increase in cross-stimulus suppression on these trials, relative to trials where the presented stimuli were indirectly associated in both memory 1 and memory 2 (Figure 5A). Thus, an *increase* in this cross-stimulus suppression measure provided a proxy for an *increase* in neural memory interference. Using this measure of memory interference, we assessed evidence for the predicted two-way relationship between the drop in concentration of relative GABA, neural memory interference, and behavioral memory interference.

First, we considered the relationship between the drop in relative GABA during application of anodal tDCS and the increase in neural memory interference from the first to the second fMRI scan task block. Across participants, the drop in relative GABA positively predicted the increase in neural memory interference measured using cross-stimulus suppression across memory 1 and memory 2 (r_17_ = 0.55, p = 0.021, Figure 5B, after accounting for changes in glutamate, see STAR Methods). Notably, cross-stimulus suppression measured from participants with minimal change in the concentration of relative GABA provided effective parametric control for participants where a larger drop in relative GABA was observed, mitigating the need for a separate sham condition. Thus, the variation in the drop in relative GABA observed across participants provided a stringent framework in which to assess the effect of EI imbalance on cross-stimulus suppression. The positive correlation between the drop in GABA and the increase in neural memory interference observed across the group suggests that interference between overlapping memories is predicted by EI imbalance.

Second, we considered the relationship between neural and behavioral memory interference. Taking behavioral performance from the surprise memory test performed after the scan, we predicted a positive relationship between neural memory interference and the percentage of foil errors on the memory test, but a negative relationship between neural memory interference and overall accuracy on the memory test. Consistent with these predictions, our cross-stimulus suppression index for neural memory interference (block 2 − block 1) positively predicted the percentage of foil errors and negatively predicted overall behavioral memory accuracy (fMRI versus foil errors: r_17_ = 0.58, p = 0.013, Figure 5C; fMRI versus overall accuracy: r_17_ = −0.67, p = 0.003, Figure 5D; after accounting for differences in learning and changes in relative GABA and glutamate, see STAR Methods). In summary, participants who showed greater cross-stimulus suppression during periods of EI imbalance also made more errors.

Together, these results suggest that a reduction in neocortical GABAergic tone leads to an increase in neural memory interference, which manifests in behavior as an increase in memory errors. While this two-way relationship capitalizes on the variability observed across participants, we next asked whether there was a main effect of anodal tDCS on neural memory interference. Using cross-stimulus suppression as a proxy for memory interference, we predicted an overall increase in neocortical memory interference during the application of anodal tDCS. Furthermore, given that a reduction in neocortical GABA resulted in pronounced re-expression of more recent associations in memory 2 (Figures S4E–S4G), we predicted memory interference would manifest in memory 1 due to expression of associations in memory 2 intruding or overriding the appropriate expression of associations in memory 1.

To maximize sensitivity to the effect of anodal tDCS, we tested for memory interference using cross-stimulus suppression within an ROI defined from the peak anodal tDCS electrode location, averaged across all participants (Figure 4C; see STAR Methods). Within this ROI, across both memory 1 and memory 2, we observed a trend toward an increase in cross-stimulus suppression during application of anodal tDCS (block 2 > block 1, t_23_ = 1.79, p = 0.087, Figure S6A). However, consistent with our prediction, for memory 1, but not memory 2, there was a pronounced increase in our cross-stimulus suppression measure of memory interference (block 2 > block 1, memory 1: t_23_ = 3.05, p = 0.006 Figures 5E, 5F, and S6B; memory 2: t_23_ = 0.57, p = 0.573, Figure S6C). To confirm that memory interference was observed during application of anodal tDCS, we also assessed effects in block 2 alone. Within the same ROI we again observed significant cross-stimulus suppression for memory 1 but not memory 2 (memory 1: t_23_ = 3.00, p = 0.006, Figures 5E–5G and S6B) and within a 10mm sphere centered on the peak of the average anodal tDCS electrode location (memory 1: t_23_ = 3.60, p = 0.027, peak-level FWE corrected using SVC with ROI shown in Figure 4D). Critically, this cross-stimulus measure of interference in memory 1 was anatomically proximal to the re-expression of directly associated memories in memory 2 reported previously (Figure 5H).

**Figure 6:**
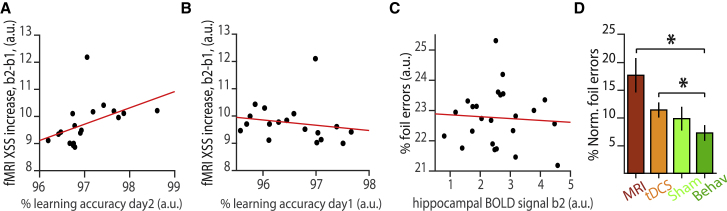
Relating Learning Accuracy to Neural Measures of Memory Interference In all panels, “a.u.” refers to “arbitrary units”; XSS indicates cross-stimulus suppression; “b” indicates block of fMRI acquisition, as shown in Figure 1F. (A) Across participants, learning accuracy for memory 2 (on day 2) positively predicted the cross-stimulus suppression index for neural memory interference (Spearman correlation: r_17_ = 0.68, p = 0.003). (B) Across participants, learning accuracy for memory 1 (on day 1) showed a negative trend with the cross-stimulus suppression index for neural memory interference (Spearman correlation: r_17_ = −0.47, p = 0.053). (C) During application of anodal tDCS in the second scan task (block 2, “b2”), the hippocampal BOLD response to trials where there was opportunity for memory interference did not predict behavioral measures of memory interference (Pearson correlation: r_23_ = −0.06, p = 0.764). (D) Both groups of participants (see STAR Methods) who received tDCS (“MRI” and “tDCS”) showed higher normalized foil errors on the surprise memory test on day 3, relative to participants who received no intervention (“Behav”) (two-sample t test: t_44_ = 2.89, p = 0.006) (shown: mean ± SEM). However, there was no difference in the percentage of foil errors made by participants who received tDCS and “Sham” (two-sample t test: t_38_ = 0.66, p = 0.515). Normalized foil errors were defined as the percentage of foil errors on foil trials, after subtracting the percentage of non-foil errors on foil trials.

These results suggest that re-expression of directly associated stimuli in memory 2 leads to interference with overlapping but contextually distinct associations in memory 1. In a final analysis, we asked whether the differential strength of memory 1 and memory 2 at encoding also predicts neural memory interference during periods of EI imbalance. We found that participants’ average learning accuracy for associations in memory 2 positively predicted the cross-stimulus suppression measure for memory interference (r_17_ = 0.68, p = 0.003, Figure 6A, after accounting for differences in learning on day 1, memory accuracy and changes in relative GABA and glutamate; see STAR Methods), while a trend toward the reverse relationship was observed for memory 1 (r_17_ = −0.47, p = 0.053, Figure 6B, after accounting for differences in learning on day 2, memory accuracy and changes in relative GABA and glutamate; see STAR Methods). Therefore, participants that weakly encoded memory 1 but strongly encoded memory 2 were more prone to memory interference. Together with results above, this suggests that interference between two memories during periods of EI imbalance can be predicted by the extent to which EI imbalance is induced (Figure 5B), but also the relative strength of the memories at encoding (Figures 6A and 6B).

### The Interplay between the Hippocampus and aLOC

These data suggest that, in addition to hippocampal pattern separation mechanisms, neocortical inhibition plays a key role in protecting memories from interference. To assess the interplay between the hippocampal and neocortical mechanisms, we reconsidered the relationship between our neural and behavioral measures of memory interference. We noted that behavioral performance on the surprise memory test after the scan session was predicted by both the hippocampal BOLD signal prior to anodal tDCS (Figure 2H) and the change in neocortical cross-stimulus suppression observed during anodal tDCS (Figures 5C and 5D). We asked whether hippocampal BOLD *during* anodal tDCS (block 2) also predicted behavioral performance. Unlike hippocampal BOLD prior to anodal tDCS (block 1), we observed no relationship between hippocampal BOLD during tDCS (block 2) and behavioral performance (hippocampal BOLD block 2 versus foil errors: r_23_ = −0.06, p = 0.764, Figure 6C, after accounting for differences in learning). Furthermore, this correlation between block 2 hippocampal BOLD and behavior was significantly different from the correlation observed between block 1 hippocampal BOLD and behavior (difference in correlation coefficient, block 1 versus block 2, permutation test: p = 0.032, Figure S6D; see STAR Methods).

While this difference in the relationship between behavior and hippocampal BOLD signal in block 1 versus block 2 may be explained by the effect of time, this seems unlikely as there was no significant change in the magnitude of the hippocampal BOLD signal from block 1 to block 2 (paired t test: t_23_ = 0.25, p = 0.802). Rather, these results suggest that, in the absence of brain stimulation, the degree to which irrelevant associative memories are represented in the hippocampus predicts memory interference. But, when neocortical GABAergic tone is reduced, signatures of neural memory interference in aLOC but not hippocampus predict memory interference. The hippocampus and aLOC thus appear to employ distinct mechanisms to mediate memory interference.

### Inducing Behavioral Memory Interference Using Brain Stimulation

In a final set of experiments, we asked whether application of anodal tDCS alone might be sufficient to induce behavioral measures of memory interference. To this end, we repeated the experiment in three additional groups of participants: (2) anodal tDCS or (3) sham-anodal tDCS (delivered using a double-blind set-up, see STAR Methods), or (4) no intervention. These three additional groups of participants performed the same set of tasks as participants receiving MRI (group 1), but outside the scanner. On the day 3 surprise memory test, we observed a significant difference between groups in mean accuracy and in the percentage of normalized foil errors using a one-way ANOVA (mean accuracy: F_82_ = 6.54, p < 0.001, Figure S1E; normalized foil errors: F_82_ = 4.39, p = 0.007, Figure 6D), and a significant effect of stimulation when using multiple regression to control for variation in learning accuracy and gender that occurred by chance across the four experimental groups (effect of stimulation on mean accuracy: t_81_ = 2.96, p = 0.004; normalized foil errors: t_81_ = 2.87, p = 0.005). Post-hoc t tests revealed a significant difference between participants who received both anodal tDCS and MRI compared to participants who did not receive any intervention (group 1, “MRI,” versus group 4, “Behav,” foil errors: t_44_ = 2.89, p = 0.006; group 2, “tDCS,” versus group 4, “Behav,” foil errors: t_38_ = 2.30, p = 0.027; Figure 6D). For participants who received sham-stimulation, there was no significant difference in behavioral performance compared to participants who did not receive any intervention (group 3, “Sham,” versus group 4, “Behav,” foil errors: t_38_ = 1.07, p = 0.292; Figure 6D). While these results suggest that anodal tDCS increased memory interference at the behavioral level, there was notably no significant difference in behavioral performance between participants who received anodal tDCS and those who received sham stimulation (group 2, “tDCS,” versus group 3, “Sham”: foil errors: t_38_ = 0.66, p = 0.515; Figure 6D). Similar results were obtained when using multiple regression to assess differences in the percentage of foil errors while controlling for variation in learning accuracy and gender that occurred by chance across the experimental groups (“MRI” versus “Behav”: t_41_ = 2.93, p = 0.005; “tDCS” versus “Behav”: t_35_ = 2.32, p = 0.026; “Sham” versus “Behav”: t_35_ = 1.38, p = 0.177; “tDCS” versus “Sham”: t_35_ = 0.59, p = 0.559), and when assessing differences in overall memory accuracy (Figure S1E). These results suggest that while anodal tDCS can induce behavioral memory interference, the expectation of anodal tDCS has a similar effect on some participants. Therefore, rather than mere application of brain stimulation, the change in the concentration of relative GABA and neural measures of memory interference are necessary to reliably predict behavioral measures of memory interference.

## Discussion

Our past experiences often overlap in their content but can nevertheless be selectively recalled without interference from other memories. Here, we investigated the neural mechanisms that help protect memories from interference. By training human participants to encode two context-dependent overlapping memories, memory 1 and memory 2, we reveal evidence for two distinct neural mechanisms that help mitigate memory interference. The first mechanism involves the hippocampus, where overlapping but context-dependent memories are pattern separated according to their relational overlap. The second mechanism involves neocortical inhibition, which protects against unwanted co-activation between neocortical representations. We discuss these two mechanisms in turn, before considering how they may interact.

In the hippocampus we observed an increase in hippocampal BOLD signal when participants observed pairs of stimuli that had different relative positions across memory 1 and 2. In the absence of brain stimulation this BOLD signal predicted participants’ performance on a surprise memory test completed after the scan. When we investigated the nature of the underlying hippocampal representations, we found evidence for pattern separation by context, where representations of stimuli were more similar to representations of other stimuli within the same memory compared to representations of other stimuli in the alternative memory. This finding is in agreement with a large body of evidence suggesting an important role for the hippocampus in pattern separation (Yassa and Stark, 2011), a mechanism that is likely mediated by orthogonal contextual representations (Butterly et al., 2012, McKenzie et al., 2014).

But, in addition, we show that pattern separation appears to be organized according to the relational overlap between memory 1 and memory 2, as representations of stimuli 3 and 6 were more dissimilar between memory 1 and memory 2, compared to all other stimuli. Therefore, pattern separation is enhanced for elements that change relational position between competing memories. These results suggest that contextual representations, which emerge from a competitive pattern separation mechanism, may be analogous to a series of cognitive maps, where each set of learned relationships is stored in a unique map distorted by the relational overlap with competing maps. Interestingly, this account is consistent with the idea that the hippocampus represents a successor representation where stimuli that predict different future states have more distinct representations (Dayan, 1993, Stachenfeld et al., 2017, Garvert et al., 2017, Momennejad et al., 2017). In this light, contextual representations within the hippocampus may be construed as configurable representations (Nadel, 2008), where the same machinery responsible for coding spatial relationships is employed when representing abstract, non-spatial stimuli.

While the hippocampus may help minimize interference by separating context-dependent memories according to their relational overlap, the sensory neocortex appears to employ a different mechanism. By downregulating the concentration of neocortical GABA using anodal tDCS (Barron et al., 2016a, Kim et al., 2014, Stagg et al., 2009), here we show that during periods of EI imbalance, neocortical memory interference increases. To quantify neural memory interference, we used ultra-high field 7T MRI to measure cross-stimulus suppression, a proxy for representational similarity between different elements of the memories (Barron et al., 2016b, Krekelberg et al., 2006). We show that the drop in GABA quantified using MRS predicts our neural measure of memory interference, which, in turn, predicts behavioral measures of memory interference. This two-way relationship reveals a key role for neocortical inhibition in protecting against memory interference.

We note that in this study we used tDCS as a *tool* to induce variability in the concentration of GABA in aLOC, which our MRS measures show was successfully achieved. Indeed, the range in inter-subject variability in induced EI imbalance provided a stringent framework within which to test our hypotheses, as fMRI measures from participants with a low change in GABA parametrically controlled for fMRI measures from participants with a higher change in GABA. The variability in GABA thus mitigated the need for a sham control group, or a control voxel from which to measure MRS, and provided a precise prediction for neural and, in turn, behavioral memory interference. When we assessed the effect of tDCS alone on behavioral memory interference, no significant difference in behavioral measures of memory interference was observed between a group of participants receiving tDCS relative to a group receiving sham stimulation. These behavioral findings confirm that a measure of EI imbalance, not mere application of tDCS, is necessary to provide a reliable predictor of memory interference.

While our findings suggest that neocortical inhibition, or inhibitory engrams, are critical for stable memory storage, they also raise a number of questions regarding the formation of inhibitory engrams and the accompanying timescale of this process. In the rodent primary auditory cortex, changes in the strength of excitatory connections are accompanied by inhibitory rebalancing after approximately 90 min (Froemke et al., 2007). This implies that a “critical period” of EI imbalance and memory instability occurs between initial learning and the formation of inhibitory engrams. Consistent with this hypothesis, a transient period of memory instability has been reported immediately after learning, during which memories may be integrated with existing knowledge that share abstract or higher-level properties (Mosha and Robertson, 2016). This integration is facilitated by offline reactivation and coordinated interactions between hippocampal and neocortical engrams (Mosha and Robertson, 2016, Preston and Eichenbaum, 2013, Schlichting and Preston, 2014).

While this opportunity to integrate newly encoded memories with existing knowledge has clear advantages, the relative instability of memories during this critical period makes them vulnerable to interference. This trade-off between integration and interference may determine the transient nature of the critical period. Indeed, if sufficient time is left between acquisition of memories that are overlapping or share a common structure, integration is no longer observed (Mosha and Robertson, 2016), nor is interference, as shown here in the absence of anodal tDCS stimulation. In addition to time, other factors such as overlearning also appear to terminate the critical period (Mosha and Robertson, 2016) by restoring EI balance with a shift from glutamate-dominated excitation to GABA-dominated inhibition (Shibata et al., 2017).

But, in addition to the timescale of inhibitory engram formation, both memory expression and memory interference are also likely to be affected by the underlying strength of the encoded memory. For example, if the excitatory strength of an associative memory is weak, neural and behavioral expression of a memory during recall will be reduced. Here, we show a significant reduction in behavioral measures of memory accuracy for associations in memory 1 relative to memory 2 (Figure 2E), even for associations that remain the same across memory 1 and 2 (Figure S4I). At the neural level, a relative weakening in the strength of excitatory connections in memory 1 relative memory 2 may explain why cross-stimulus suppression between directly associated stimuli, an index for memory expression, was only observed for memory 2 (Figures S4D and S4E), while memory interference effects were only observed for memory 1 (Figures 5E–5G).

By combining ultra-high field 7T fMRI with MRS, brain stimulation, and behavioral measures, the protocol described here illustrates how macroscopic measures of the human brain can be used to index micro-circuit processes. This has notable translational value for clinical populations where microcircuit disruption is not readily amenable to investigation, particularly conditions that have been attributed to disturbances in EI balance. For example, in schizophrenia delusions and hallucinations have been attributed to perturbed inhibitory gating (Vogels and Abbott, 2007, Yizhar et al., 2011), while memory loss and confusion in early stage dementia have been associated with hyperactivity (Busche and Konnerth, 2016). The data presented here may be considered a model for these clinical phenotypes, where neocortical EI imbalance causes unwanted reactivation of irrelevant memories that may have overlapping excitatory engrams with those activated by incoming stimuli. In the absence of appropriate inhibitory regulation, otherwise latent memory engrams are activated in an uncontrolled manner causing confusion or hallucinations. The protocol implemented here thus provides a basis from which to further explore mechanisms responsible for memory impairment in clinical conditions that report evidence for EI imbalance.

Finally, we considered the interplay between the hippocampal and neocortical mechanisms for mitigating against memory interference. When we reduced the concentration of neocortical GABA using anodal tDCS, interestingly, the hippocampal BOLD signal no longer predicted behavioral performance on the surprise memory test. This suggests that inhibitory gating in the aLOC may influence context-dependent hippocampal representations. Although beyond the scope of this investigation, it is also interesting to speculate about the reverse relationship, the influence of context-dependent relational hippocampal codes on neocortical memory engrams. One possibility is that analogous to cholinergic modulation of neocortical interneurons observed during context-dependent behavior (Kuchibhotla et al., 2017), the hippocampus mediates selective release of neocortical memory engrams by targeting neocortical inhibition (Barron et al., 2017). This interaction between the hippocampus and neocortex may facilitate selective reactivation of neocortical representations during memory recall, providing an index for distributed memory engrams (Teyler and Rudy, 2007).

## STAR★Methods

### Key Resources Table

**Table:** 

REAGENT or RESOURCE	SOURCE	IDENTIFIER
**Software and Algorithms**		

MATLAB 2016b	Mathworks	https://www.mathworks.com
Psychtoolbox-3	Psychtoolbox developers	http://psychtoolbox.org
SPM12	FIL Methods group	http://www.fil.ion.ucl.ac.uk/spm
LCModel	Provencher, 1993	http://s-provencher.com
RSA Toolbox	Nili et al., 2014	http://www.mrc-cbu.cam.ac.uk/methods-and-resources/toolboxes/
ROAST	Huang et al., 2017	https://www.parralab.org/roast/

**Supporting Data and Code**		

Scripts and data for reproducing all figures	Github	https://github.com/rskool/meminf/RK_00001

### Contact for Resource Sharing

Further information and requests for resources should be directed to and will be fulfilled by the lead contact, Dr. Helen Barron (helen.barron@pharm.ox.ac.uk).

### Experimental Model and Subject Details

#### Participants

91 healthy volunteers participated in the study (group 1: “MRI” with tDCS, n = 30, mean age of 24.0, 17 females; group 2: “tDCS,” n = 20, mean age of 21.9, 18 females; group 3: “Sham,” n = 20, mean age of 24.1, 9 females; group 4: “Behav,” n = 21, mean age of 23.2, 11 females). All experiments were approved by the Oxford University ethics committee (reference number ref R43594/RE001). All participants gave informed written consent.

In group 1, three participants dropped out after the first day as they were not able to achieve the day 1 training criteria (see below). In group 1, one participant was excluded due to a fault on the scanner which prevented data acquisition in the second half of the scan session. In group 4, one participant was excluded after revealing that they had an arteriovenous malformation in the cerebellum.

### Method Details

#### Behavioral training

All behavioral tasks were coded in MATLAB 2016b using Psychtoolbox (version 3.0.13). Seven different stimuli were presented to the participant, referred to as 1:7. Stimuli were rotationally invariant gray shapes (Figure 1A), which were observed in one of four possible rotations, with each rotation separated by 90° (as described in Barron et al., 2016a). Learned associations between these rotating shapes are known to be represented in a localized and superficial region of neocortex (Barron et al., 2016a), thus providing a suitable target for anodal tDCS (see below). The experiment was conducted across three days (Figure 1F). On the first day participants performed a *training task*, see below, to learn seven bidirectional associations between the seven stimuli. The set of associations could be arranged in a ring structure (Figure 1B), where each stimulus was associated with two other stimuli (1 with 2, 2 with 3, etc. and 7 with 1). Participants were not explicitly made aware of the ring structure. Stimulus allocation within the ring structure was randomized across participants using MATLAB’s random number generator.

The *training task* included two phases. During phase 1 of the training task participants were passively exposed to seven pairs of associated stimuli. On each trial of phase 1, a pair of associated stimuli was presented against a background color (blue or yellow, depending on the training day) for 3 s duration (see Figures S1A and S1B). The stimulus presented on the left hand-side was randomized on each trial. Each pair of stimuli was presented four times in total, once for each of the four possible orientations of each stimulus. Across trials, the order in which pairs of stimuli were presented was randomized. The background color was different on day 1 and day 2, thus providing a contextual cue for the learned associations. Participants were allowed to repeat phase 1 of the training task before each phase 2 block if they wished to do so.

During phase 2 of the training task participants performed an active task, involving a three-alternative forced-choice task (Figures 1D and 1E). On each trial of the three-alternative forced-choice task, one of the seven stimuli was shown as a probe stimulus for 1 s before three choice stimuli were presented in randomized positions across the screen. As in phase 1, stimuli were presented against a background color (blue or yellow, depending on the training day), used to provide a contextual cue. The three choice stimuli included one stimulus to which the probe stimulus was associated, and two stimuli to which the probe stimulus was not associated. Participants were instructed to select the correctly paired stimulus as fast as possible, without compromising their accuracy, using the appropriate keyboard button, ‘b’, ‘n, or ‘m’. If participants failed to make a response within 3 s they received an on-screen message indicating that they were too slow. Participants received feedback for each choice, where the probe stimulus together with the correctly paired choice stimulus was presented for 1.5 s. For each correct response, participants were assigned 50p. Each task block included 100 trials in total, and each pair of associated stimuli was presented at least 14 times. Across trials, the rotation of the presented stimuli and the trial order were randomized. At the end of each task block three percent of trials were randomly selected and participants received the sum total reward from these trials.

On the second day, participants again learned seven associations between the seven stimuli, however the position of the stimuli within the implicit ring structure was altered relative to the first day. In particular, stimuli ‘3′ and ‘6’ were switched, resulting in four new associations and three associations that remained the same across days (Figure 1C). To indicate this change in the implicit associative structure, the background color of the screen (blue or yellow) was changed from day 1 to day 2. The color assigned to day 1 and 2 was randomized across participants. To learn the new arrangement of stimuli, participants underwent both phase 1 and 2 of the training task again, thus following the same protocol as used on day 1 (Figures 1E and S1B).

On both day 1 and 2, the criterion for stopping the phase 2 training task was as follows: participants were required to complete at least five blocks and reach at least 90% accuracy on all of the seven associations. If after five blocks of phase 2 of the training task participants did not reach the criterion of 90% accuracy on all of the seven associations, then they were required to continue completing phase 2 task blocks until this criterion was met (Figure S1F). By ensuring that participants completed at least five blocks of phase 2 of the training task on both day 1 and 2, our experimental protocol minimized differences in the number of phase 2 trials completed on day 1 compared to day 2, and minimized differences in the number of phase 2 trials completed across participants. Across all 4 experimental groups, 3 out of 91 participants dropped out of the experiment after not reaching 90% accuracy on all seven associations despite completing multiple phase 2 training task blocks. Data from these participants was not included in any analyses. As some participants got tired during the final training blocks of the phase 3 training task, performance during the final and/or penultimate task block was sometimes compromised. For this reason, ‘learning accuracy’ on both day 1 and 2 was estimated as the average performance across trials on each participant’s highest performing task block (Figures S1C and S1D).

On the third day of the experiment, participants were required to perform the fMRI scan task (see below, Figure 1G). The fMRI scan task was performed inside the scanner for group 1, but outside the scanner for groups 2-4. Immediately after exiting the scanner (group 1), or immediately after the scan task (groups 2-4), participants were given a surprise memory test designed to assess participants’ memory for the associations learned on both day 1 and day 2 (Figure 2D). The memory test involved a variant of the three-alternative forced-choice task used during training on day 1 and 2 (Figures 1D and 1E). However, unlike the training task, the background color switched randomly between trials to indicate either the day 1 or day 2 context, and the task was presented in the absence of feedback. Given the probe stimulus and the background color, participants were instructed to select the correct associated stimulus. The memory test constituted 100 trials, with half presented on the yellow background and half on the blue background.

#### fMRI scan task

The *fMRI scan task* involved participants viewing the seven visual stimuli used in the training task (1:7), presented via a computer monitor, which for group 1 was then projected onto a screen inside the scanner bore. On each trial two stimuli were presented consecutively for 800 ms each, with an inter-stimulus interval of 300 ms (Figure 1G). The inter-trial interval was selected from a truncated gamma distribution with mean of 2.9 s, minimum of 1.5 s and maximum of 9.7 s. To control for potential confounding effects of expectation suppression (Summerfield et al., 2008), all stimuli, all possible pairs of non-repeating stimuli, and all possible rotations of each stimulus were presented equally often in a fully randomized order. Participants were required to perform a task incidental to the contrast of interest which involved identifying whether the presented stimuli were familiar or “oddball.” Oddball stimuli, defined as stimuli that did not belong to the training set 1 to 7, were randomly inserted into 7% of trials. Participants were instructed to press a button on an MR compatible button box using their right index finger when they identified “oddball” stimuli but not if both stimuli on the trial were familiar. No feedback was given. Each task block lasted twenty minutes and included 196 trials, with each stimulus presented 52 times, with 13 examples of each of the four possible rotations per stimulus. Within each block, each pair of non-repeating stimuli was presented 4 times in each context, while each pair of repeating stimuli was presented 2 times in each context. Each participant performed two task blocks.

#### fMRI imaging protocol

Participants in group 1 completed the scan task within a 7 Tesla Magnetom MRI scanner (Siemens) with 1-channel transmit and a 32-channel phased-array head coil (Nova Medical, USA) at the Wellcome Centre for Integrative Neuroimaging Centre (University of Oxford). Current 7T radio-frequency (RF) coil designs suffer from B1 inhomogeneity effects which were pronounced in the right temporal lobe. To overcome this, we positioned two barium titanate dielectric pads (4:1 ratio of BaTiO3:D2O, with a relative permittivity of around:300, and size 110 × 110 × 5 mm^3^) over the right temporal lobe in all 7T scanning sessions, causing a “hotspot” in the RF distribution at the expense of distal regions (Brink and Webb, 2014, Teeuwisse et al., 2012). The tDCS electrode was situated between the dielectric pad and the head.

To acquire fMRI data a multiband echo planar imaging (EPI) sequence was used to acquire 50 1.5 mm thick transverse slices with 1.5 mm gap, in-plane resolution of 1.5 × 1.5 mm^2^, repetition time (TR) = 1.512 s, echo time (TE) = 20 ms, flip angle = 85°, field of view 192 mm, and acceleration factor of two. To increase SNR in brain regions for which we had strong prior hypotheses, we restricted the fMRI sequence to a partial volume, thus increasing the number of measurements acquired. The partial volume covered occipital and temporal cortices (see Figures S2A–S2C) and in each session 644–723 volumes were collected (:20 min). For each participant, a T1-weighted structural image was acquired to correct for geometric distortions and perform co-registration between EPIs, consisting of 176 0.7 mm axial slices, in-plane resolution of 0.7 × 0.7 mm^2^, TR = 2.2 s, TE = 2.96 ms, and field of view = 224 mm. A field map with dual echo-time images was also acquired (TE1 = 4.08 ms, TE2 = 5.1 ms, whole-brain coverage, voxel size 2 × 2 × 2 mm^3^).

#### MRS

For participants in group 1, during the scan session MRS data was acquired as described in (Barron et al., 2016a). B0 shimming was performed in a two-step process. First, GRE-SHIM (field of view, 384 × 384 mm^2^; TR = 600 ms; TE1/2 = 2.04/4.08 ms; slice thickness 4 mm; flip angle 15°; slices 64; scan time 45 s) was used to determine the optimal first- and second-order shim currents. The second step involved only fine adjustment of first-order shims using FASTMAP (Gruetter and Tkác, 2000). The modified semi-LASER sequence, previously shown to have minimal chemical shift displacement error (CSDE), was used with TE = 36 ms, TR = 5–6 s to acquire MRS measurements in a 2 × 2 × 2 cm^3^ volume of interest (VOI), positioned next to the tDCS electrode (Figure 4C) (Oz and Tkáč, 2011).

For each MRS measurement between 65 and 130 scan averages were collected, giving a total acquisition time of around 10 min. Three measurements were acquired for each participant, before and during tDCS, and after the second task block (Figure 1F). Metabolites were quantified using LCModel (for example spectra: Figures S3A and S3B) (Provencher, 1993, Provencher, 2001). The model spectra of alanine (Ala), aspartate (Asp), ascorbate/vitamin C (Asc), glycerophosphocholine (GPC), phosphocholine (PCho), creatine (Cr), phosphocreatine (PCr), GABA, glucose (Glc), glutamine (Gln), glutamate (Glu), glutathione (GSH), myo-inositol (myo-Ins), Lactate, N-acetylaspartate (NAA), N-acetylaspartylglutamate (NAAG), phosphoethanolamine (PE), scyllo-inositol (scyllo-Ins) and taurine (Tau) were generated based on previously reported chemical shifts and coupling constants by VeSPA Project (Versatile Simulation, Pulses and Analysis) (Govindaraju et al., 2000, Tkac, 2008).

The unsuppressed water signal acquired from the VOI was used to remove residual eddy current effects and to reconstruct the phased array spectra (Natt et al., 2005). To improve comparability across spectra, the water component of the spectra was then removed before single scan spectra were summed from 32 channels, corrected for frequency and phase variations induced by participants’ motion, and then summed. LCModel analysis was performed on all spectra within the chemical shift range 0.5 to 4.2 ppm (Provencher, 1993).

Reliable LCModel fits were achieved in 20 of the 26 participants and metabolite concentration relative to total Creatine concentration were estimated, relative to unsuppressed water spectrum acquired from the same VOI. In the remaining 6 participants the relative GABA quantification was either unreliable or inestimable due to lipid contamination and broader linewidth. The lipid contamination could be observed directly in the spectral range 1.9-0.5 ppm (Figures S3A and S3B). The broader linewidth, quantified using Full-Width at Half Maximum (FWHM), was significantly higher in these six participants relative to the 20 participants included for analysis (two sample t test: t_24_ = 3.73, p = 0.001, Figure S3C). Participants with inestimable GABA were excluded from all data analyses that included MRS data.

All measured metabolites included in the analysis had Cramér–Rao lower bound (CRLB) values ≤ 50% (Bednařík et al., 2015). Relative to baseline concentrations (‘Before tDCS’), the change in relative GABA (Figure 4E), relative glutamate (Figure 4F), and other metabolite concentrations (Table S1) were compared across conditions using a two-tailed paired t test where the direction of the effect was unknown and a one-tailed paired t test in instances where the direction of the effect was predicted from previous data (i.e., for the change in relative GABA). Thus, all t tests were performed using within-subject comparisons.

#### tDCS

Immediately before and during Block 2 of the scan task, participants in groups 1, 2 and 3 received tDCS using a DC-Stimulator (Eldith) which delivered a 1 mA current to the brain. For group 1, the current was delivered while participants were inside the 7T MRI scanner. For groups 2 and 3, the current was delivered outsider the scanner using a double-blind procedure (see below). To ensure that the tDCS was suitable for use in the 7T scanner, we used two 5 × 7 cm^2^ MRI compatible electrodes (Easycap) fitted with 5 kOhm resistors to minimize the risk of heating or eddy current induction. Using high-chloride EEG electrode gel (Easycap) as a conducting paste, the anodal electrode was placed on the scalp above the region of right temporal cortex previously identified as encoding the association between paired shapes (Figures 4A–4B), approximately at the 10–20 T6 node location. The cathodal electrode was placed over the contralateral supraorbital ridge (Figures 4A and S5). For participants in group 1, a cod-liver oil capsule was taped to the anodal electrode, immediately underneath the resistor, to make the electrode MR-visible and allow for its location to be mapped onto the anatomical brain surface (Figure 4C). For all participants, the impedance of tDCS was checked prior to stimulation. In group 1, this impedance check was performed before participants entered the scanner and again once the participant was lying inside the bore of the magnet with extension leads connected to the stimulator. For participants in group 1 and 2, tDCS was delivered using a 10 s ramp-up of the current, which was then held at 1 mA current for a total of 20 min, before a 10 s ramp-down. For participants in group 3, sham stimulation involved mimicking the prickling sensation of stimulation using a 10 s ramp-up of current, turning stimulation off for 20 minutes and then repeating the 10 s ramp-up. For participants in all groups 1-3, the stimulation protocol commenced 10 min prior to the start of the second fMRI scan task (Figure 1F). At the end of the experiment, participants in groups 2 and 3 were debriefed: they were informed that they may have received sham stimulation and were asked to declare whether they believed they had received real or sham stimulation. The blinded researcher (R.K., see below) also declared whether they believed the participants had received real or sham stimulation.

#### Double-blind procedure for anodal/sham tDCS

Participants in groups 2 and 3 were first recruited, before being randomly assigned to the anodal (group 2) or sham (group 3) stimulation condition using a random number generator. Randomization was performed by a researcher (H.B.) who was not involved in recruitment. Behavioral training, electrode placement, the scan tasks, surprise memory test and debrief were carried out by a researcher blind to the stimulation condition (R.K.). tDCS was delivered by a researcher who was aware of the stimulation conditions and who was not involved in any of the behavioral training or assessment (H.B.). Analysis of participants responses during the debrief indicated that 55% of participants in group 2 (‘tDCS’) and 75% of participants in group 3 (‘sham’) believed they received anodal tDCS stimulation. The blinded researcher believed that 55% of participants in group 2 (‘tDCS’) and 45% of participants in group 3 (‘sham’) received anodal tDCS stimulation.

#### fMRI data analysis

For all MRI datasets obtained from participants in group 1, pre-processing was carried out using SPM12 (https://www.fil.ion.ucl.ac.uk/spm/). Two participants were excluded from the fMRI analysis due to poor performance on the fMRI scan task (< 80% accuracy on one or more of the two task blocks), suggesting that they may have fallen asleep during the task. For the remaining 24 participants images were corrected for signal bias, realigned to the first volume, corrected for distortion using field maps, normalized to a standard EPI template and smoothed using an 8-mm full-width at half maximum Gaussian kernel. To remove low frequency noise from the preprocessed data, a high-pass filter was applied to the data using SPM12′s default settings. For each participant and for each scanning block, the resulting fMRI data was analyzed in an event-related manner using two different general linear models (GLMs), one designed for univariate analyses and a second designed for multivariate analyses. In both GLMs explanatory variables used a delta function to indicate the onset of a trial and were then convolved with the hemodynamic response function.

The first GLM, used to analyze univariate BOLD effects, was applied to data from each of the two scan task blocks separately, and to data from both scan task blocks together. In the design, a total of 46 different explanatory variables were included per block. 42 of these explanatory variables were included to account for each possible pair of visual stimuli (‘1’ and ‘2′, ‘1’ and ‘3′ etc.) in each of the two background contexts (i.e., memory 1 or memory 2), regardless of the order in which the two stimuli were presented within the pair. An additional 4 explanatory variables were used to model trials that included repeating stimuli or trials were ‘odd-ball’ stimuli had been presented in each of the two background contexts (i.e., memory 1 or memory 2). Finally, for each task block an additional 6 scan-to-scan motion parameters produced during realignment were included in the GLM as additional nuisance explanatory variables to account for motion-related artifacts.

Using the output of this first GLM for the univariate analysis, the following three principal contrasts of interest were assessed. First, to measure cross-stimulus adaptation as an index for expression of directly associated stimuli (Figure S4), the contrast of interest involved comparing fMRI BOLD signal for trials with pairs of stimuli separated by more than one link across both memories (‘unassociated’; i.e., memory 1 and memory 2 links 2-7, 5-7, 2-4, 1-5, 1-4, 2-5, 4-7, 3-6, 1-6, 1-3) with fMRI BOLD signal for trials with pairs of stimuli separated by one link in both memories (‘associated’; i.e., memory 1 and memory 2 links 1-2,4-5,7-1). Second, to measure cross-stimulus adaptation as an index for memory interference (Figures 5 and 6A–6B), the contrast of interest involved comparing fMRI BOLD signal for pairs of stimuli separated by more than one link across both memories (‘unassociated’; i.e., memory 1 and memory 2 links 2-7, 5-7, 2-4, 1-5, 1-4, 2-5, 4-7, 3-6, 1-6, 1-3) with fMRI BOLD signal for pairs of stimuli separated by more than one link in the current context, but only one link in the alternative context (‘hidden’; i.e., memory 1: links 3-5, 4-6, 2-6, 3-7; memory 2: links 3-4, 5-6, 2-3, 6-7). Third, to measure the BOLD response to trials where there was an opportunity for memory interference (Figures 2A–2C, 2H, and 6C), the contrast of interest involved comparing fMRI BOLD signal for pairs of stimuli that shared the same topological relationship across the two memories (‘stable’, i.e., links that did not include stimuli 3 or 6) with fMRI BOLD signal for pairs of stimuli that had a different topological relationship across the two memories (‘unstable’; i.e., links that included stimuli 3 or 6).

### Quantification and Statistical Analysis

#### fMRI statistics and ROI specification

From the first GLM, the contrast images of all participants were entered into a second-level random effects analysis. To test for fMRI cross-stimulus suppression effects in an unbiased fashion, parameter estimates obtained from the relevant GLM were extracted from an independent region of interest (ROI) (see below). Paired t tests were used to assess differences in the main effect between sessions. When testing evidence for replication of our previous findings (Barron et al., 2016a) a one-tailed test was used. In all other instances, two-tailed tests were used. The significance level was set to p < 0.05.

To assess fMRI cross-stimulus suppression effects in the neocortex, three ROIs were defined. To assess evidence for replication of previously published results (Figure S4), an independent ROI was defined using the previously published dataset, after thresholding the contrast of interest at p < 0.01 uncorrected (Figures 4B and S4B) (Barron et al., 2016a). To assess evidence for memory interference, an ROI was defined from the peak average location of the anodal tDCS electrode. This was estimated using the T1 scan to identify the location of the cod-liver oil capsule taped immediately underneath the resistor of the anodal electrode. For each participant, the ventral-dorsal coordinate was taken from the upper edge of the cod-liver capsule. The medial-lateral coordinate was projected 20mm from the lateral surface, consistent with the peak medial-lateral coordinate of previously published cross-stimulus suppression induced by application of tDCS (Figure 4B) (Barron et al., 2016a). For each participant, an 8mm sphere was then drawn around the identified coordinate, and the sphere was warped to a standard EPI template. Across individuals the peak average ROI was calculated (Figure 4C). To perform SVC for multiple comparisons, a 10mm sphere was drawn around the peak of the group average tDCS electrode location (Figure 4D) and statistical significance assessed using peak-level FWE correction at p < 0.05. Capitalizing on variance across participants within this larger ROI, the extracted fMRI cross-stimulus suppression measures were correlated with changes in GABA and behavior (Figure 5B–5D).

To assess changes in BOLD signal in the hippocampus (Figure 2), an anatomical hippocampal mask was used to perform SVC for multiple comparisons (Figure 2G), with peak-level FWE correction at p < 0.05.

#### Representational Similarity Analysis

The second GLM was used to assess multivariate effects. In this GLM each trial was modeled as a unique explanatory variable. All trials across Block 1 and 2 were included. In addition, 6 scan-to-scan motion parameters produced during realignment were included in the GLM as additional nuisance explanatory variables to account for motion-related artifacts. The output of this GLM was used to estimate the representational similarity between each trial, using the representational similarity analysis toolbox (RSA) (Kriegeskorte et al., 2008, Nili et al., 2014). The dissimilarity between the response pattern elicited on each trial was estimated using the Mahalanobis distance (Walther et al., 2016), and expressed using correlation distances (1-r). For each participant, the dissimilarity value for the response patterns associated with each trial were represented in each cell of a representational dissimilarity matrix (RDM). Thus, for each stimulus, all trials containing the stimulus were included to estimate a stimulus representation, e.g., trials contributing to the representation of stimulus 1 in memory 1 included all pairs of stimuli shown on a yellow background that included stimulus 1, i.e., 1-1, 1-2, … 1-7. To estimate summary statistics, the Kendall rank correlation coefficient was estimated between the participant’s RDM and a model RDM (Figures S2D–S2F). These summary statistics were then tested at the group level using a two-sided Wilcoxon signed-rank test across participants. This indicated whether the difference in correlation coefficients between two conditions was greater than zero. This approach allowed for significant within stimulus exemplar discrimination (Figures S2H–S2I). To estimate a confusion matrix across memory 1 and 2 (Figure 3A), the RDM for each participant was sorted by stimulus type and average representational dissimilarity measure within and between each stimulus was calculated to generate a 7x7 matrix.

#### Correlations between fMRI, behavioral and MRS data

To assess the relationship between hippocampal BOLD signal and behavior, a Pearson’s correlation was used. Due to outlier data points in the fMRI cross-stimulus suppression measure (see Figures 5B–5D, 6A–6B, and S4H), Spearman’s rank correlation was used to assess the relationship between fMRI cross-stimulus suppression and changes in GABA or behavior. Correlations were plotted between standardized residuals, using a partial correlation method to account for unwanted variance attributed to other variables, such as participants’ performance on the *training task*. The partial correlation involved using ordinary-least-squares multiple regression to estimate the residuals:

$$Y_{i}=b_{0}+b_{1}x_{i}+\varepsilon_{i}\;\mathrm{and}\;Y_{j}=c_{o}+c_{1}x_{j}+\varepsilon_{j}$$

where Y_i_ and Y_j_ represent the variables for which there is hypothesized to be a predictive relationship (e.g., neural and behavioral measures), x_i_ and x_j_ represent ‘nuisance’ variables for which variance is to be accounted (e.g., learning accuracy shown in Figures S1C and S1D), b_0_ and c_0_ represent the intercepts, b_1_ and c_1_ represent the regression coefficients on the ‘nuisance’ variables. The partial correlation coefficient was then estimated as the correlation between the resulting residuals, with the mean of the original variables added to the standardized residuals to aid interpretability: (ε_i_ + b_0_ + mean(Y_i_)) and (ε_j_ + c_0_ + mean(Y_j_)).

A permutation test was used to quantify the difference in correlation between behavioral performance and hippocampal BOLD in Block 1 versus Block 2. To estimate a null distribution subject labels for hippocampal BOLD signal were permuted 10,000 times, before being correlated with behavioral performance. The difference in correlation between the Block 1 and Block 2 correlations was then computed for all 10,000 examples. The true difference between Block 1 and Block 2 correlations was compared against the null distribution to generate a p value (Figure S6D).

### Data and Software Availability

Upon publication MATLAB scripts for reproducing all figures will be made available on GitHub (https://github.com/rskool/meminf/RK_00001). Upon publication group t-stat images, anonymized subject-specific parameter estimates extracted from ROIs, and relevant experimental parameters that support the findings of this study will be made available on GitHub (https://github.com/rskool/meminf/RK_00001). The accession number for data reported in this paper is [RK_00001].

## Supplemental Information

Document S1. Figures S1–S6 and Table S1

Document S2. Article plus Supplemental Information
