# Supplementary material for: The Hippocampus and Neocortical Inhibitory Engrams Protect against Memory Interference
Source: Neuron. 2019 Feb 6;101(3):528–541.e6. doi: 10.1016/j.neuron.2018.11.042 (PMC6560047; doi:10.1016/j.neuron.2018.11.042)
Supplement: Document S1. Figures S1–S6 and Table S1 [file mmc1.pdf]

Neuron, Volume *101*

Supplemental Information

The Hippocampus and Neocortical Inhibitory Engrams Protect against Memory Interference

Renée S. Koolschijn, Uzay E. Emir, Alexandros C. Pantelides, Hamed Nili, Timothy E.J. Behrens, and Helen C. Barron

Supplemental Information

Figures S1-S6:

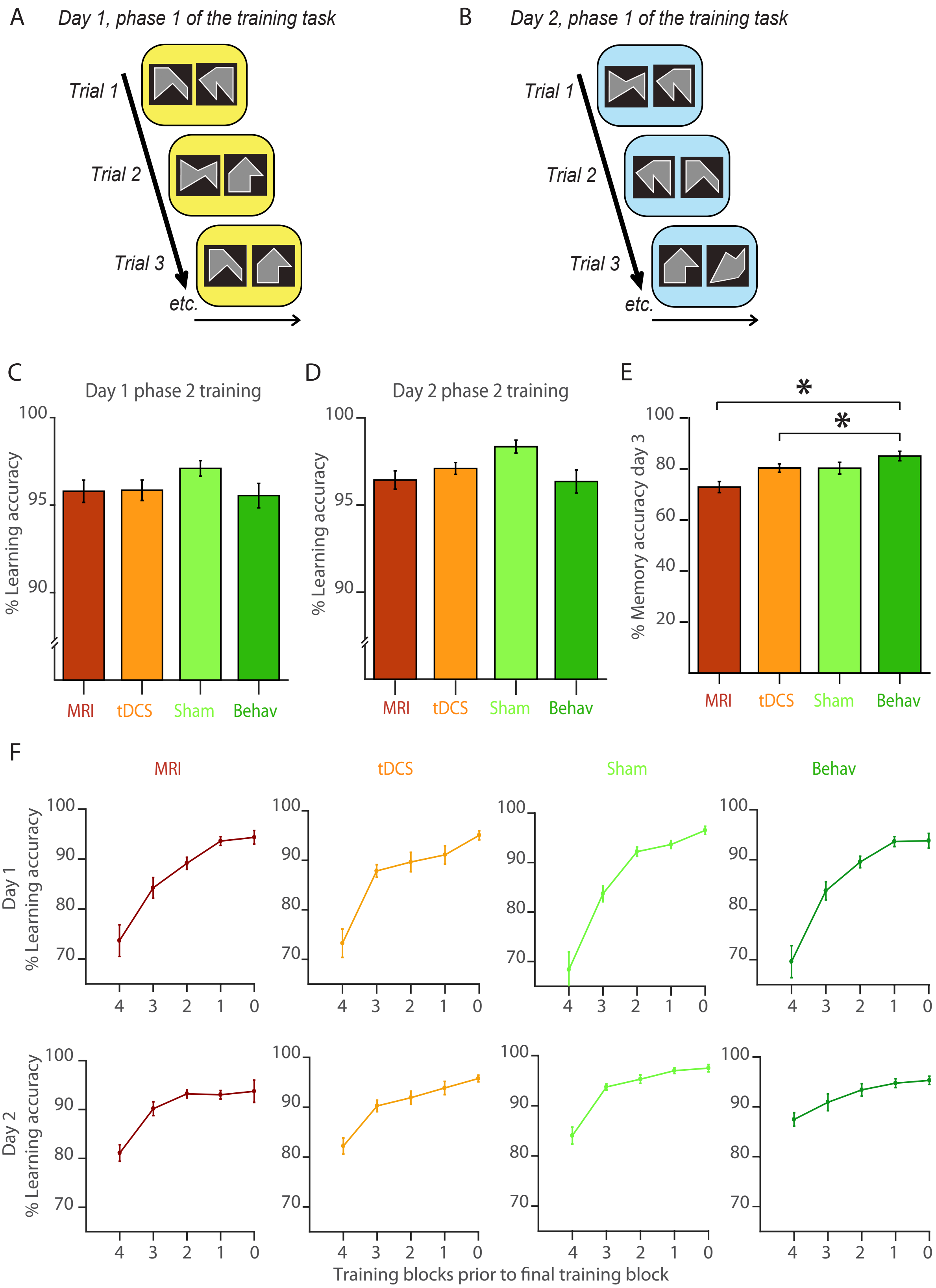

Figure S1| Training task and surprise memory test, related to Figure 1

A-B) The training task performed by participants on day 1 and 2 of the task involved two phases. Phase 1 involved a passive task (shown here) while phase 2 involved an active task (shown in Fig. 1D-E). On each trial of phase 1 of the training task participants were shown a pair of associated stimuli for 3 s duration. The stimulus that appeared on the left-hand side of the screen was randomised. Each pair of stimuli was presented 4 times in total and the order in which pairs of associated stimuli were presented was randomised across trials. The background colour on the screen provided a contextual cue for each day of the training task and was either yellow (**A**) or blue (**B**). Participants were required to passively observe the pairs of stimuli and instructed to try to learn the associative pairings. **C-D)** Percentage learning accuracy during phase 2 of the training task (Fig. 1D-E) for the four different groups of participants (see Methods) on day 1 (**C**) and day 2 (**D**). Shown: mean ±SEM. Some participants got tired during training and showed a reduction in task performance during the final and/or penultimate task block. For this reason, 'learning accuracy' was estimated as the average performance across trials on each participant's highest performing task block. On day 1 there was no significant difference in learning accuracy between any pair of the four experimental groups. However, on day 2, a significant difference in learning accuracy was observed between the 'sham' group and the 'MRI' group (t_{44}=2.12, p=0.040), but not between any other groups. This difference occurred by chance as the allocation of participants to the 'tDCS' and 'sham' group was double blinded. **E)** Accuracy on the surprise memory test on day 3 for the four different groups of participants. Shown: mean ±SEM. We observed a significant difference between groups in mean memory accuracy using a one-way ANOVA (mean accuracy: F_{82}=6.54, p<0.001), and a significant effect of stimulation when using multiple regression to control for variation in learning accuracy and gender that occurred by chance across the four experimental groups (effect of stimulation on mean accuracy: t_{81}=2.96, p=0.004). Post-hoc t-tests revealed significantly lower overall memory accuracy for participants who received tDCS and MRI relative to participants who received no intervention ('MRI' vs. 'Behav': t_{44}=4.11, p<0.001), with a similar trend for participants who received tDCS without MRI ('tDCS' vs 'Behav': t_{38}=1.91, p=0.064). However, there was no difference in performance between the 'tDCS' and 'Sham' groups ('tDCS' vs 'Sham': t_{38}=0.02, p=0.986). Similar results were obtained when using multiple regression to control for variation in learning accuracy and gender that occurred by chance between the four experimental groups ('MRI' vs 'Behav': t_{41}=5.17, p<0.001; 'tDCS' vs 'Behav': t_{35}= 2.19, p=0.035; 'tDCS' vs 'Sham': t_{35}=0.568, p=0.574). **F)** Percentage learning accuracy during phase 2 of the training task (Fig.1D-E) for the four different groups of participants (see Methods), split by training block for both day 1 (upper row) and day 2 (lower row). Shown: mean ±SEM. Performance accuracy is shown for the final 5 training blocks as all participants were trained until they completed at least 5 training blocks (see Methods).

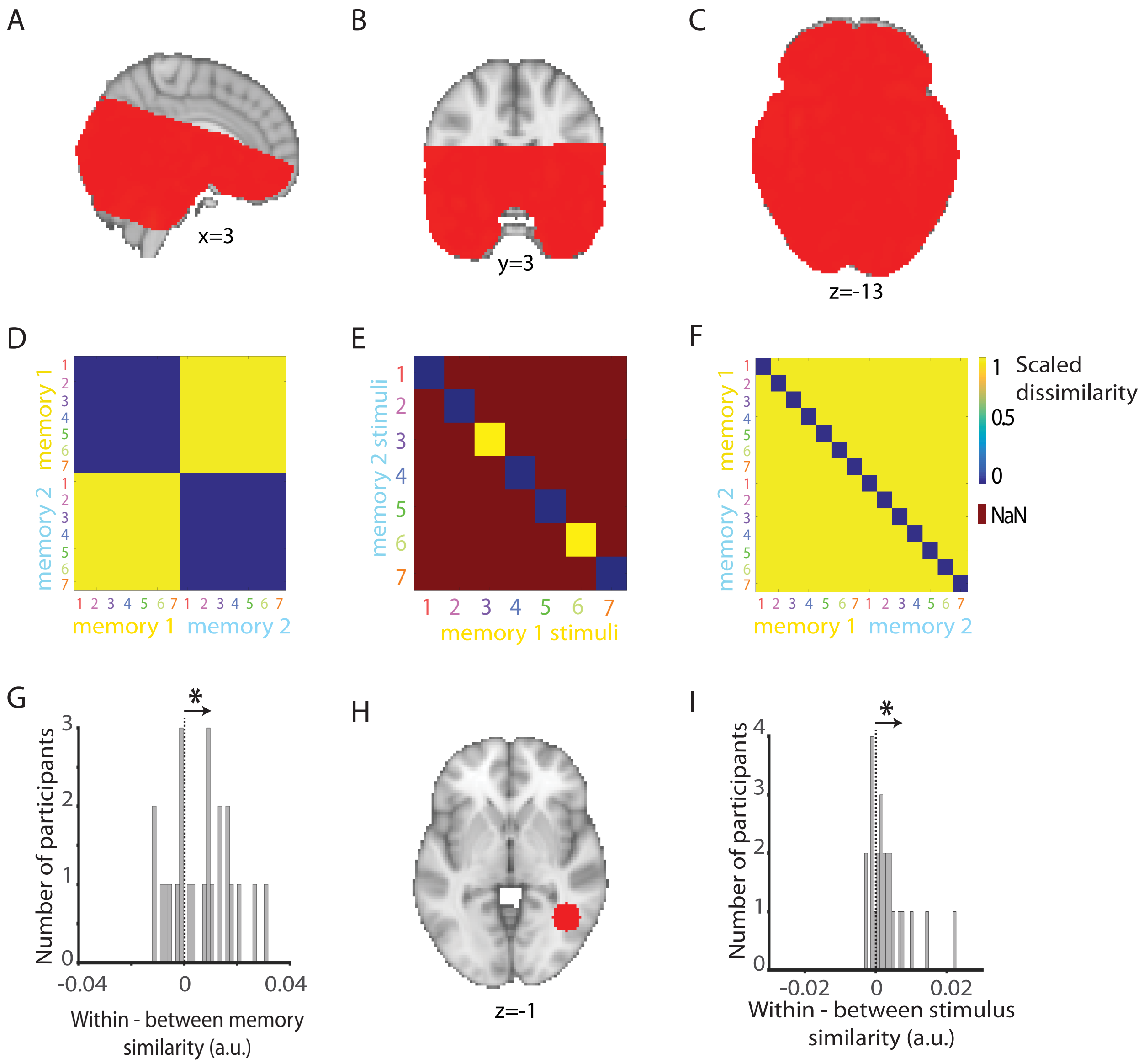

Figure S2 | Example of fMRI partial volume and RSA models and additional analyses, related to Figures 2-3
A-C) To increase SNR in brain regions for which we had strong prior hypotheses, we restricted the fMRI sequence to a partial volume, thus allowing for an increase in the number of measurements acquired due to shorter TR. The partial volume covered occipital and temporal cortices. Here, brain regions included in the partial volumes of all participants are shown for the (**A**) sagittal, (**B**) coronal and (**C**) axial plane. Orientation: neurological. **D-F)** Model representational dissimilarity matrices (RDMs) used to assess evidence for: **(D)** Within versus between memory dissimilarity, with results shown in Fig. 3B; **(E)** Between memory dissimilarity for stimuli that change their relational position across memory 1 and 2 (i.e. stimuli 3 and 6) relative to all other stimuli (1,2,4,5,7), with results shown in Fig. 3C; **(F)** Within versus between stimulus dissimilarity, with results shown in Fig. S2I. **G)** The RDM for each participant was correlated with a model RDM shown in Fig. S2D to test evidence for pattern separation of hippocampal representations by memory. All trials with either a 3 or 6 stimulus were excluded from the analysis. Across participants, significant representational similarity *within* memory 1/2 versus *between* memory 1 and 2 was observed (Wilcoxon sign rank test: Z_{23}=2.46, p=0.014). Note: the dissimilarity of a trial to itself was excluded from the analysis. 'a.u.' refers to 'arbitrary units'. **H)** ROI shown in Fig. 4D, centered on peak tDCS electrode location (see Methods). Orientation: neurological. **I)** For each participant, we assessed the dissimilarity in activity patterns between- versus within-stimulus exemplars in memory 1 and in memory 2 (e.g. [1 to 2, 1 to 3, 1 to 4, etc] minus [1 to 1, 2 to 2, 3 to 3, etc]), and observed a significant positive difference within the aLOC ROI shown in *H* (between – within stimulus exemplars: Wilcoxon sign rank test: Z_{23}=2.75, p=0.003). 'a.u.' refers to 'arbitrary units'. Note: the dissimilarity of a trial to itself was excluded from the analysis.

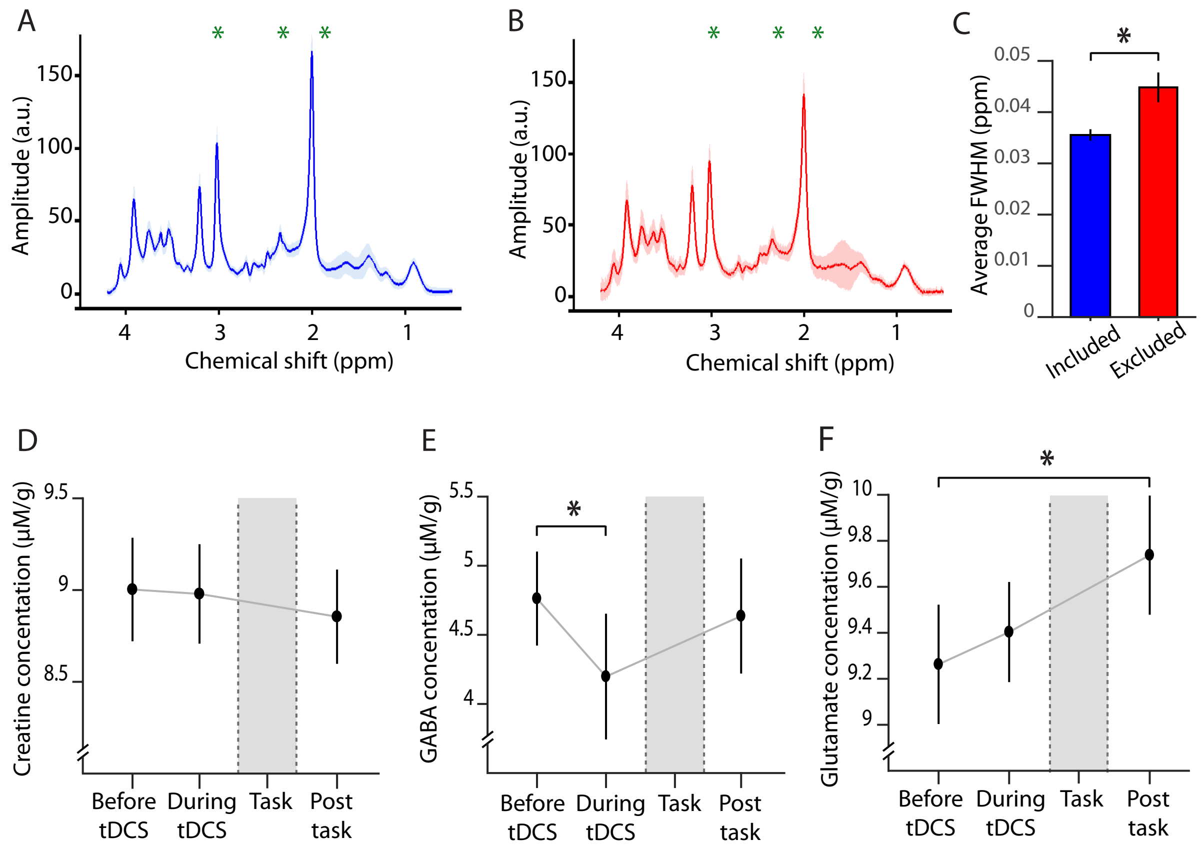

Figure S3 | MRS spectra, subject inclusion, and additional analyses, related to Figure 4

A) Average spectra for all participants data included in MRS analysis (shown: mean ±SEM). Chemical shifts of the three GABA peaks are indicated using green stars. **B)** Average spectra for those participants who were rejected from the MRS analysis (shown: mean ±SEM). Data from these participants were noisy, and had lipid contamination in the region 1.9-0.5ppm (i.e. in the region of the lowest GABA peak). This resulted in either inestimable or highly unreliable GABA estimates. Chemical shifts of the three GABA peaks are indicated using green stars. **C)** Relative to participants included in the MRS analysis (shown in *A*), those participants rejected from the MRS analysis (shown in *B*) had broader linewidth, estimated using full-width at half maximum (FWHM) using LCModel (two-sample t-test: t_{24}=3.73, p=0.001) (shown: mean ±SEM). **D)** There was no significant change in the concentration of total Creatine (Cr+PCr) across the 3 MRS measurements ('Before tDCS' – 'During', t_{19}=0.26, p=0.799; 'Before tDCS' – 'Post task' t_{19}=1.37, p=0.186; shown: mean ±SEM). **E-F)** By assessing metabolite concentrations relative to total Creatine and by comparing the concentration of GABA/glutamate between two time points in a within subject manner, our analyses (Fig. 4E-F) controlled for variation in voxel tissue and CSF in the MRS voxel used across subjects and thus mitigated the need for partial volume correction. To check that the proportion of CSF in the MRS voxel did not affect water signal referencing in LCModel we reassessed the change in the concentration of GABA and glutamate across the 3 MRS sessions, after accounting for the proportion of CSF. We show that our results shown in Fig. 4E-F remain unchanged: **E)** A significant reduction in relative GABA was observed during tDCS ('Before tDCS' – 'During tDCS', t_{19}= 2.24, p=0.019, shown: mean ±SEM); **F)** A significant increase in relative glutamate was observed after the second scan task ('Post-task' – 'Before tDCS', t_{19}= 2.56, p=0.019, shown: mean ±SEM).

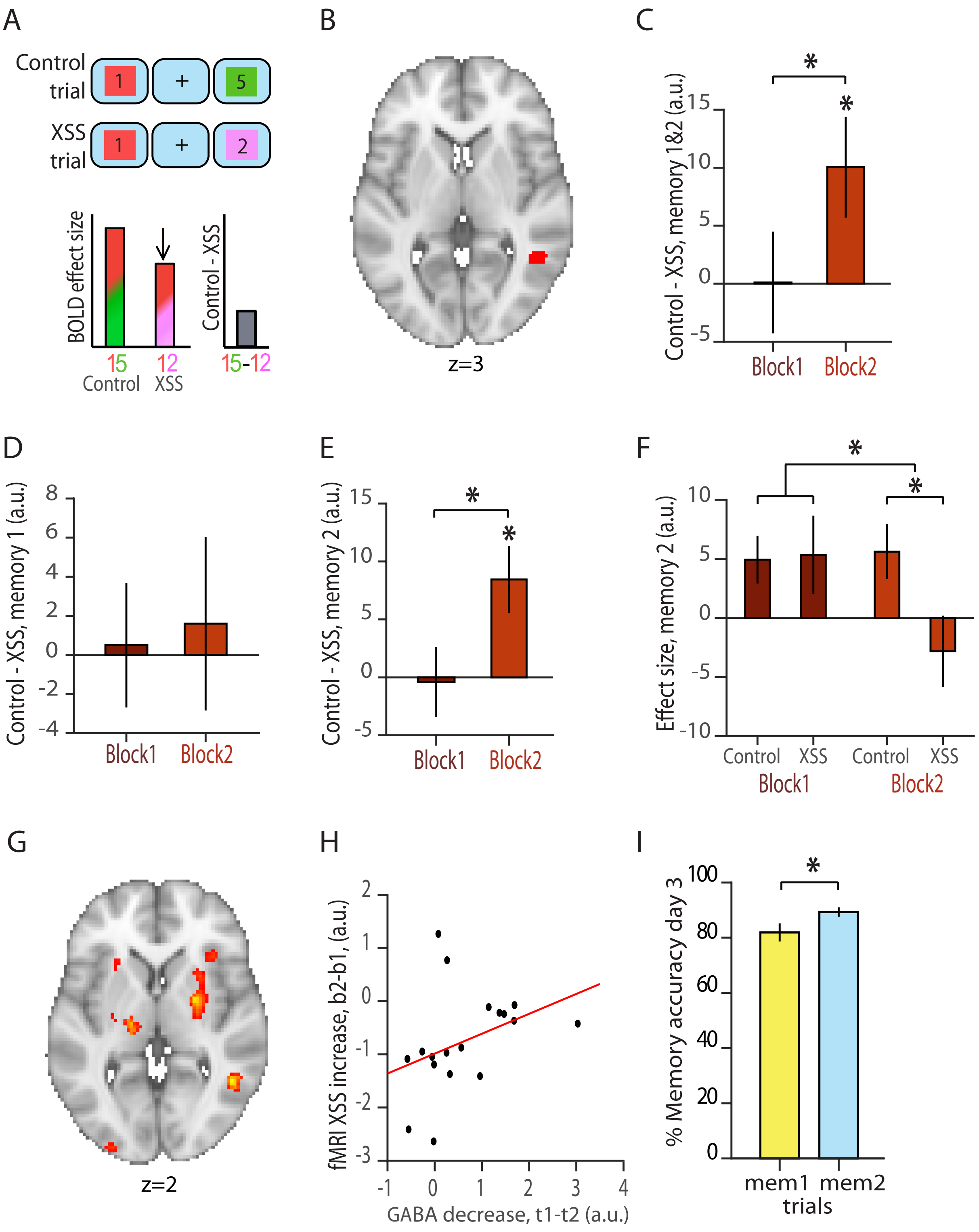
A
Control trial
1
+
5
XSS trial
1
+
2
BOLD effect size
Control - XSS
15
12
Control
XSS
15-12
B
z=3
C
Control - XSS, memory 1&2 (a.u.)
Block1
Block2
D
Control - XSS, memory 1 (a.u.)
Block1
Block2
E
Control - XSS, memory 2 (a.u.)
Block1
Block2
F
Effect size, memory 2 (a.u.)
Control
XSS
Control
XSS
Block1
Block2
G
z=2
H
fMRI XSS increase, b2-b1, (a.u.)
GABA decrease, t1-t2 (a.u.)
I
% Memory accuracy day 3
mem1
mem2
trials

Figure S4 | Replication of previous findings (Barron et al., 2016): Otherwise dormant associative memories are re-expressed during periods of EI imbalance, related to Figure 4-5

'XSS' indicates cross-stimulus suppression; *'b'* indicates block of fMRI acquisition; *'t'* indicates timepoint of MRS acquisition, as shown in Fig. 1F. **A)** When participants performed the scan task in EI imbalance, we predicted an increase in cross-stimulus suppression ('XSS') on trials where pairs of directly associated stimuli were presented (e.g. stimuli 1 and 2), relative to trials where pairs of unassociated stimuli were presented ('Control', e.g. stimuli 1 and 5). The difference between 'Control' and 'XSS' trials could be indexed using the BOLD signal to provide a measure of cross-stimulus suppression. **B)** To test replication of our previously published result (Barron et al., 2016a) (shown in Fig. 4B), we used an independently defined ROI in aLOC, taken from our previous dataset (Fig. 4B, thresholded at p<0.01, see Methods). Orientation: neurological. **C)** For directly associated stimuli across both memory 1 and 2, extracted parameter estimates (shown: mean ±SEM) revealed a significant increase in fMRI cross-stimulus suppression during brain stimulation, and significant fMRI cross-stimulus suppression during brain stimulation (within the ROI shown in *B*: 'Control' – 'XSS' for Block 2 – Block 1: t_{23}=1.73, p=0.049; for Block 2: t_{23}=2.31, p=0.015). Thus, replicating our previous findings (Barron et al., 2016a), these results show that reducing GABAergic tone increases repetition suppression between associated stimuli. **D)** Between directly associated stimuli in memory 1, extracted parameter estimates (shown: mean ±SEM) revealed no significant change in fMRI cross-stimulus suppression during brain stimulation (within the ROI shown in *B*: 'Control' – 'XSS' for Block 2 – Block 1: t_{23}=0.23, p=0.823). **E)** Between directly associated stimuli in memory 2, extracted parameter estimates (shown: mean ±SEM) revealed a significant increase in fMRI cross-stimulus suppression during brain stimulation, and significant fMRI cross-stimulus suppression during brain stimulation (within the ROI shown in *B*: 'Control' – 'XSS' for Block 2 – Block 1: t_{23}=2.29, p=0.016; Block 2: t_{23}=2.92, p=0.004). **F)** Extracted parameter estimates from *E* split into the 'Control' and 'XSS' conditions, as described in *A* (shown: mean ±SEM). **G)** T-statistic map for cross-stimulus suppression between directly associated stimuli in memory 2 during Block 2 shown in *E-F*, thresholded at p<0.01 uncorrected for visualization. Significant cross-stimulus suppression for directly associated elements of memory 2 could also be observed in Block 2 within a 10mm radius sphere defined around the peak tDCS electrode location for all participants (Fig. 4D, see Methods) (t_{23}=4.17, p=0.010, peak-level FWE corrected using SVC). Orientation: neurological. **H)** There was a significant positive correlation between the change in relative GABA ('Before tDCS' – 'During tDCS') and the increase in fMRI cross-stimulus suppression (Block 2- Block 1) observed in the ROI shown in Fig. 4D, averaged across both memory 1 and 2 (Spearman correlation: r_{17}=0.52, p=0.028, after accounting for changes in glutamate, see Methods). Thus, the decrease in relative GABA during application of anodal tDCS positively predicted the increase in cross-stimulus suppression between directly associated stimuli in memory 1 and 2. *'b'* indicates block for fMRI acquisition as shown in Fig. 1F. *'t'* indicates 'timepoint' of MRS measurement acquisition, as shown in Fig. 1F**. I)** Across memory 1 and memory 2 (Fig. 1B-C), 3 of the 7 associations remained the same: those between stimuli 1 and 2, 1 and 7 and 4 and 5. Memory accuracy on the surprise memory test (shown: mean ± SEM) for these stable associations was significantly different between memory 1 (day 1, yellow) and memory 2 (day 2, blue), with higher memory accuracy when participants recalled these associations in the more recent memory 2 (paired t-test: t_{25}=2.16, p=0.040).

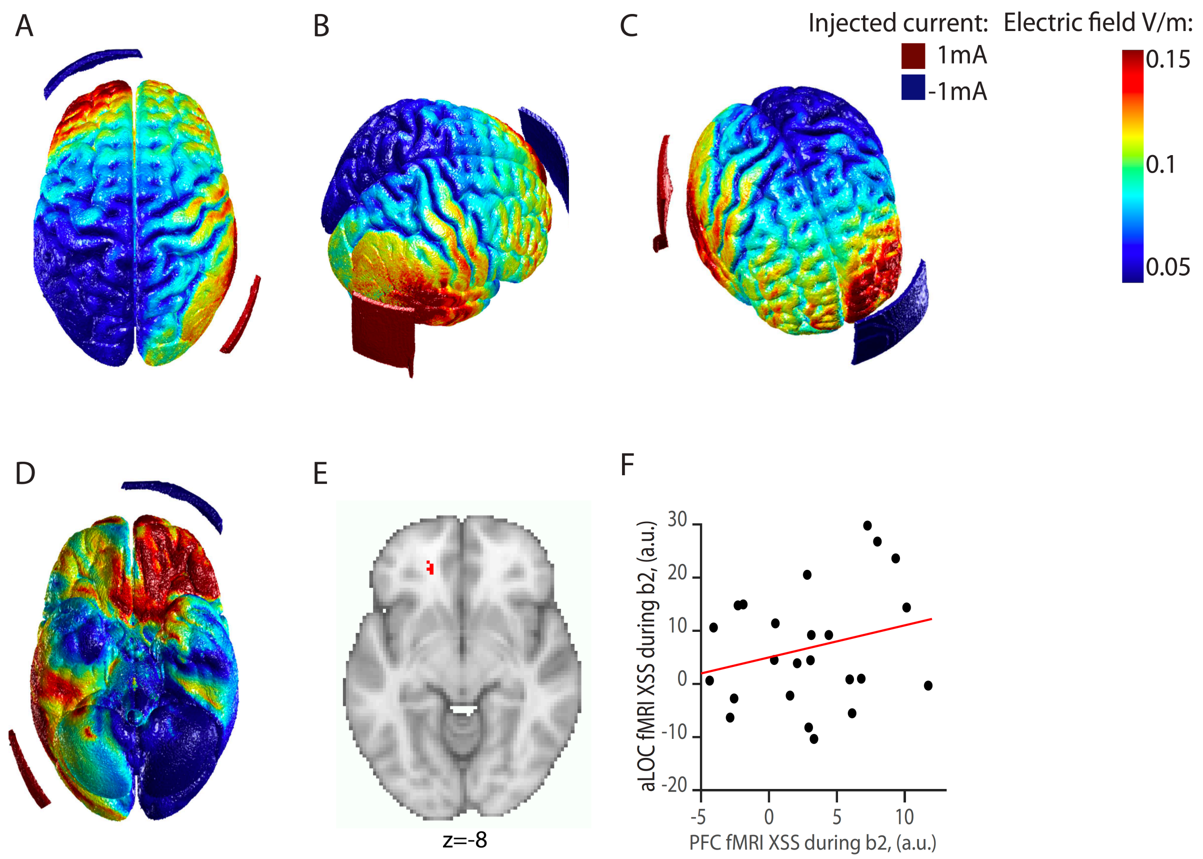

Figure S5 | Simulated effect of tDCS across the brain and effect of cathodal stimulation on left prefrontal cortex (PFC), related to Figures 4-5

A-D) Using open source software, ROAST, (Huang et al., 2017) we used a current model to estimate the electric field induced by tDCS across neocortex for our electrode configuration. To simulate the effect of the anodal electrode, a 1mA current was injected into the right aLOC. To simulate the effect of the cathodal electrode, a -1mA current injected into the left prefrontal cortex. This stimulation configuration gave rise to an elevated electric field (V/m) under both the anodal and cathodal electrodes. **(A)** Dorsal surface. **(B)** Right hand hemisphere, including region under the anodal electrode. **(C)** Anterior view, showing region under cathodal electrode. **(D)** Ventral surface. **E-F)** Our cross-stimulus suppression contrast for memory interference did not show any suprathreshold voxels (defined as p<0.001, uncorrected) underneath the cathodal electrode. This suggests that the effects reported from aLOC (Fig. 5) cannot be explained by downstream effects of cathodal stimulation near to left PFC. However, to check that *subthreshold* fMRI effects in PFC (defined as voxels surviving p<0.01 uncorrected) cannot explain the reported effects in aLOC (Fig. 5) we assessed the relationship between these two brain regions. **(E)** ROI in PFC, defined from a contrast for our cross-stimulus suppression index for memory interference, thresholded at p<0.01 uncorrected. Orientation: neurological. **(F)** The relationship between PFC and aLOC: parameter estimates were extracted from PFC (ROI shown in *E*) and from aLOC (peak average tDCS electrode location shown in Fig. 4C) during Block 2 of the fMRI scan task and the cross-stimulus suppression index for memory interference was assessed. Cross-stimulus suppression in PFC did not predict the measure for memory interference in aLOC (r_{23}=0.25, p=0.240). This suggests that cross-stimulus suppression effects reported in aLOC (Fig. 5) cannot be explained by downstream effects of cathodal stimulation near to left PFC. 'XSS' indicates cross-stimulus suppression; '*b*' indicates block for fMRI acquisition, as shown in Fig. 1F**.**

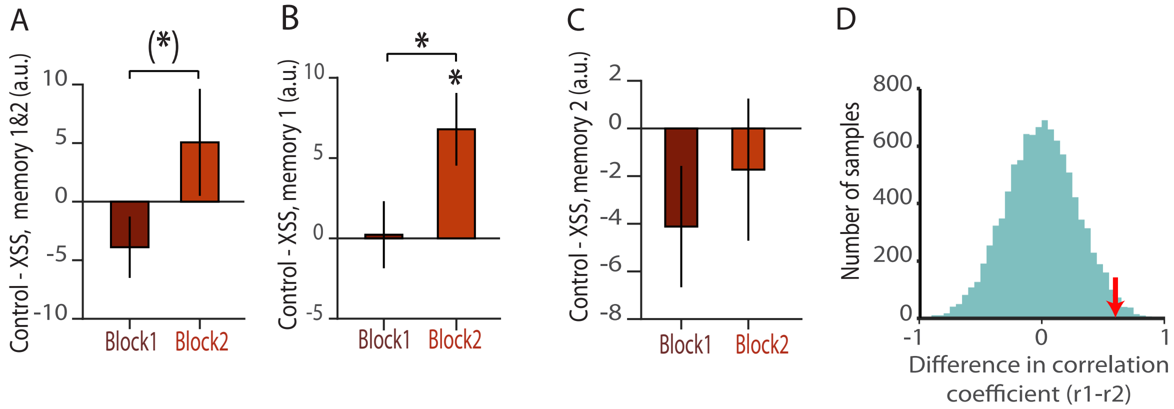

Figure S6 | Memory interference effects in memory 1 and 2, related to Figures 5-6

A) Cross-stimulus suppression was used to index the change in memory interference before and during application of tDCS, as shown in Fig. 5. Across both memory 1 and 2 there was a trend towards an increase in the cross-stimulus suppression index for memory interference (paired t-test: t_{23}=1.79, p=0.087) (shown: mean ±SEM). **B)** As shown in Fig. 5E: within an ROI defined from the peak average tDCS electrode location shown in Fig. 4C, extracted parameter estimates for memory 1 (shown: mean ±SEM) revealed a significant increase in the fMRI cross-stimulus suppression measure for memory interference ('Control' – 'XSS', as shown in Fig. 5A) from Block 1 to 2 and during Block 2 alone ('Control' – 'XSS' for Block 2 – Block 1: t_{23}=3.05, p=0.006; 'Control' – 'XSS' for Block 2: t_{23}=3.00, p=0.006). **C)** Unlike for memory 1 shown in *B* and Fig. 5E-F, no significant change in the fMRI cross-stimulus suppression measure for memory interference was observed for memory 2 (t_{23}=0.57, p=0.573, shown: mean ±SEM). **D)** During Block 1 but not Block 2 of the scan task hippocampal BOLD predicted subsequent behavioural performance on the surprise memory test (Fig. 2H and Fig. 6C). To assess the significance of the difference in correlation coefficient, a null distribution of 10,000 samples was estimated using a permutation test (see Methods). Here, the null distribution can be observed in green and the difference in correlation between block 1 and 2 is indicated by the red arrow.

Table S1:

Metabolite	Before tDCS	During tDCS	Post-task
Alanine	1.39 ± 0.42	1.52 ± 0.44	1.31 ± 0.45
Ascorbate	1.11 ± 0.17	1.29 ± 0.18	1.22 ± 0.17
Aspartate	3.18 ± 0.34	2.78 ± 0.34	2.26 ± 0.27
Glycerophosphorylcholine	0.93 ± 0.07	0.86 ± 0.08	0.94 ± 0.09
Phosphorylcholine	0.75 ± 0.06	0.83 ± 0.06	0.76 ± 0.07
Creatine	4.96 ± 0.14	4.97 ± 0.15	4.91 ± 0.14
Phosphocreatine	3.04 ± 0.14	3.03 ± 0.15	3.09 ± 0.14
GABA	4.77 ± 0.34	4.18 ± 0.45	4.53 ± 0.39
Glucose	2.22 ± 0.18	2.31 ± 0.21	2.31 ± 0.15
Glutamine	7.30 ± 0.37	7.36 ± 0.42	7.04 ± 0.36
Glutamate	9.26 ± 0.26	9.39 ± 0.22	9.75 ± 0.26
Glutathione	0.89 ± 0.08	0.77 ± 0.11	0.79 ± 0.10
Inositol	7.27 ± 0.15	7.19 ± 0.14	7.19 ± 0.16
Lactate	1.10 ± 0.20	1.18 ± 0.15	0.89 ± 0.14
N-acetylaspartate (NAA)	13.25 ± 0.37	13.19 ± 0.34	13.28 ± 0.39
N-acetylaspartylglutamate (NAAG)	2.34 ± 0.11	2.29 ± 0.12	2.31 ± 0.10
Phosphoethanolamine	1.91 ± 0.17	1.97 ± 0.15	1.95 ± 0.19
Scyllo-Inositol	0.18 ± 0.03	0.17 ± 0.03	0.17 ± 0.03
Taurine	0.65 ± 0.08	0.53 ± 0.08	0.51 ± 0.07

Table S1 | Average concentration of all metabolites measured using MRS, related to Figure 4
For each metabolite, the concentration was measured relative to Creatine and then averaged across participants (shown: mean, ± SEM). As reported in the main text, a significant decrease in the concentration of GABA was observed during tDCS, and a significant increase in the concentration of glutamate observed after the second task block (Fig. 4E-F). Of the other metabolites measured (n=17), only one showed a significant change in concentration across the three MRS measurements: the concentration of aspartate significantly decreased after the second task block ('before tDCS' – 'post-task', t_{19}=4.29, p<0.001).
